# Cryptic species in a well-known habitat: applying taxonomics to the amphipod genus *Epimeria* (Crustacea, Peracarida)

**DOI:** 10.1038/s41598-018-25225-x

**Published:** 2018-05-02

**Authors:** Jan Beermann, Michael V. Westbury, Michael Hofreiter, Leon Hilgers, Fabian Deister, Hermann Neumann, Michael J. Raupach

**Affiliations:** 1 Alfred Wegener Institute Helmholtz Centre for Polar and Marine Research, Department of Functional Ecology, PO Box 120161, 27515 Bremerhaven, Germany; 2 Alfred Wegener Institute Helmholtz Centre for Polar and Marine Research, Biologische Anstalt Helgoland, Helgoland, Germany; 3 Helmholtz Institute for Functional Marine Biodiversity, Oldenburg, Germany; 4 University of Potsdam, Institute for Biochemistry and Biology, Karl-Liebknecht-Str. 24-25, 14476 Potsdam, Germany; 5 Museum für Naturkunde, Leibniz Institute for Evolution and Biodiversity Science, Invalidenstr. 43, 10115 Berlin, Germany; 6 Carl von Ossietzky University Oldenburg, Institute for Biology and Environmental Sciences, PO Box 2503, 26111 Oldenburg, Germany; 7 Senckenberg am Meer, Department for Marine Research, Südstrand 40, 26382 Wilhelmshaven, Germany; 8 Senckenberg am Meer, German Center of Marine Biodiversity (DZMB), Südstrand 44, 26382 Wilhelmshaven, Germany

## Abstract

Taxonomy plays a central role in biological sciences. It provides a communication system for scientists as it aims to enable correct identification of the studied organisms. As a consequence, species descriptions should seek to include as much available information as possible at species level to follow an integrative concept of ‘taxonomics’. Here, we describe the cryptic species *Epimeria frankei* sp. nov. from the North Sea, and also redescribe its sister species, *Epimeria cornigera*. The morphological information obtained is substantiated by DNA barcodes and complete nuclear 18S rRNA gene sequences. In addition, we provide, for the first time, full mitochondrial genome data as part of a metazoan species description for a holotype, as well as the neotype. This study represents the first successful implementation of the recently proposed concept of taxonomics, using data from high-throughput technologies for integrative taxonomic studies, allowing the highest level of confidence for both biodiversity and ecological research.

## Introduction

The biological discipline of taxonomy formally describes, classifies and names extant and extinct species^1^. It is therefore of central importance within the biological sciences, providing a communication system for scientists of all biological disciplines^2^. This is of pivotal importance as results and conclusions of studies that rely on incorrect identification and/or classification of the analysed organisms are highly questionable^3^. Despite all efforts in the description of organisms over the past centuries, many species remain to be discovered, classified, described, and named. It has been estimated that 3 to 100 million species exist on Earth^4–7^ with the most recent suggestions narrowing the extant diversity of life to approx. 8.7 million species^8^. These numbers clearly give the impression that still only a small fraction of all species on Earth (about 2 millions, with a rate of approx. 20,000 new species descriptions per year; see http://www.esf.edu/species/) have been scientifically described so far^8^.

Traditionally, morphological characteristics such as size, shape, colour, and anatomical structures are used to classify and describe species. With the rise of molecular biology in the middle of the 20^th^ century, various new technologies have become available for taxonomic studies, such as the analysis of size, shape and the number of chromosomes^9^ or allozyme and isozyme electrophoretic analyses^10,11^. The development of the polymerase chain reaction (PCR) offered a broad variety of methods for sequence analyses of DNA^12^, including the study of random amplified polymorphic DNA fragments (RAPDs)^13–15^ and PCR-restriction fragment length polymorphisms (PCR-RFLPs)^16,17^. However, the continuous improvement of sequencing technologies and their decreasing costs promoted the application of nucleotide sequences in taxonomy. Various nuclear and particularly mitochondrial markers are commonly in use today. Due to their faster evolutionary rates (compared to most nuclear genes), mitochondrial protein-coding genes, and here in particular the cytochrome *c* oxidase subunit I (CO1), are regarded as powerful taxonomic markers for genetic diversity analysis at less inclusive levels, including families, genera and species^18^. Especially the 5′ end of CO1 has become extremely popular as it is flanked by two “universal” primer regions which allow successful amplification of a broad range of metazoans^19–21^. Therefore, this approximately 650 base-pair fragment was proposed as global standard for species identification - the so-called “barcode region” for animals^22,23^. Although different issues potentially affect the applicability of DNA barcodes and mitochondrial DNA in general for species identification (e.g., hybridizations, incomplete lineage sorting or the presence of mitochondrial pseudogenes)^24^, many studies successfully demonstrated its usefulness to delineate and identify species across a broad range of taxa^25–30^. Consequently, many recently published species descriptions include DNA barcode sequence data^31–34^. The application of high-throughput sequencing technologies allows even more detailed characterizations of species, particularly by incorporating complete genome sequence data^35^, as it has already been successfully implemented for a number of Bacteria and Archaea^36–38^.

Due to their high taxonomic diversity, ecological significance and many different life styles across aquatic and terrestrial environments, amphipod crustaceans represent excellent model organisms to study speciation and radiation of organisms. To date, more than 10,000 amphipod species have been described^39^, but various taxonomic uncertainties still remain, for example in the species-rich genus *Gammarus*^40–42^, the *Tryphosa*-group^43^, or in the subterranean genus *Niphargus*^44^. Additionally, recent studies indicate that species diversity of the Amphipoda is even larger than it had been expected based on the use of “traditional” morphological methods alone^45–47^.

Here we describe a cryptic species in the amphipod genus *Epimeria* from the North Sea, *Epimeria frankei* sp. nov. and also redescribe its sister species, *Epimeria cornigera* (Fabricius, 1779). Both descriptions include comprehensive morphological information, DNA barcodes, and complete nuclear 18S rRNA gene sequences. In addition, we provide full mitochondrial genome information for the first time for a holotype and a neotype within the Metazoa. This previously unmatched level of information for a taxonomic description represents the first implementation of the recently proposed concept of taxonomics, using comprehensive data based on modern molecular technologies for taxonomic studies^35,48^.

## Results

### DNA barcodes

A total number of 86 DNA barcodes of *Epimeria cornigera* (*n* = 23), *Epimeria frankei* (*n* = 57), and *Gammarus locusta* (*n* = 6) were analysed. For the analysed specimens of the genus *Epimeria*, the barcode fragment length ranged from a minimum of 616 to the full fragment length of 658 bp, including four barcode fragments with intermediate lengths (*n* = 4, 4.9%). The mean nucleotide frequencies were A = 0.29, C = 0.21, G = 0.16, and T = 0.34 for *Epimeria cornigera*, and A = 0.28, C = 0.22, G = 0.16, and T = 0.34 for *Epimeria frankei*. Intraspecific K2P distances ranged from 0% to 0.65% for *Epimeria cornigera* and 0% to 3.16% for *Epimeria frankei*. Interspecific distances between both species had values from 8.69% to 11.81% (Table 1). *Epimeria cornigera* had one unique BIN (AAU2062), whereas two BINs were identified for *Epimeria frankei* (AAU2061, ACS7228) (Table 1).

**Figure 1:**
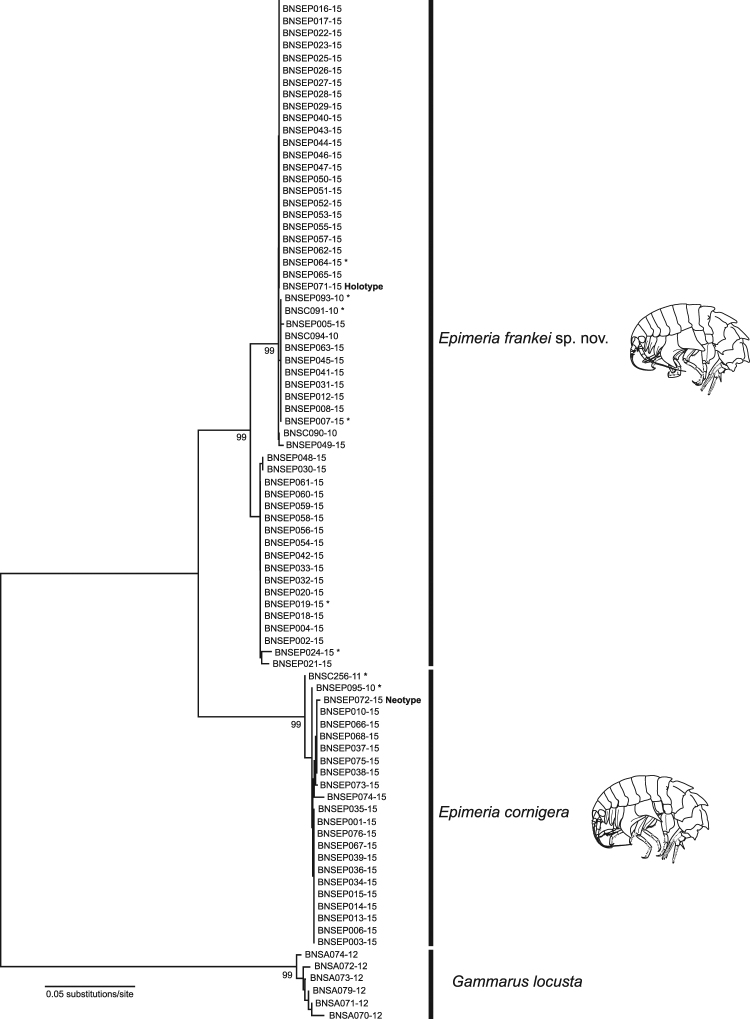
Neighbor-joining phylogenetic tree of the analysed COI sequences of *Epimeria cornigera*, *Epimeria frankei* sp. nov., and *Gammarus locusta* as outgroup. The optimal tree with the sum of branch length = 0.44256318 is shown. Evolutionary distances were computed using the Kimura 2-parameter model for substitution. Numbers next to internal branches are non-parametric bootstrap values (in %). Only values above 90% are shown. Asterisks indicate specimens with additional complete 18S rRNA gene sequence data. All codon positions were included. All ambiguous positions were removed for each sequence pair. There were a total of 658 positions in the final dataset.

The NJ analyses based on K2P distances showed distinct clusters with bootstrap support values of 99% for all three species (Fig. 1). In the case of *Epimeria frankei*, our analysis revealed two distinct lineages with a minimum K2P distance of 2.17% and each corresponding to a distinct BIN.

**Figure 2:**
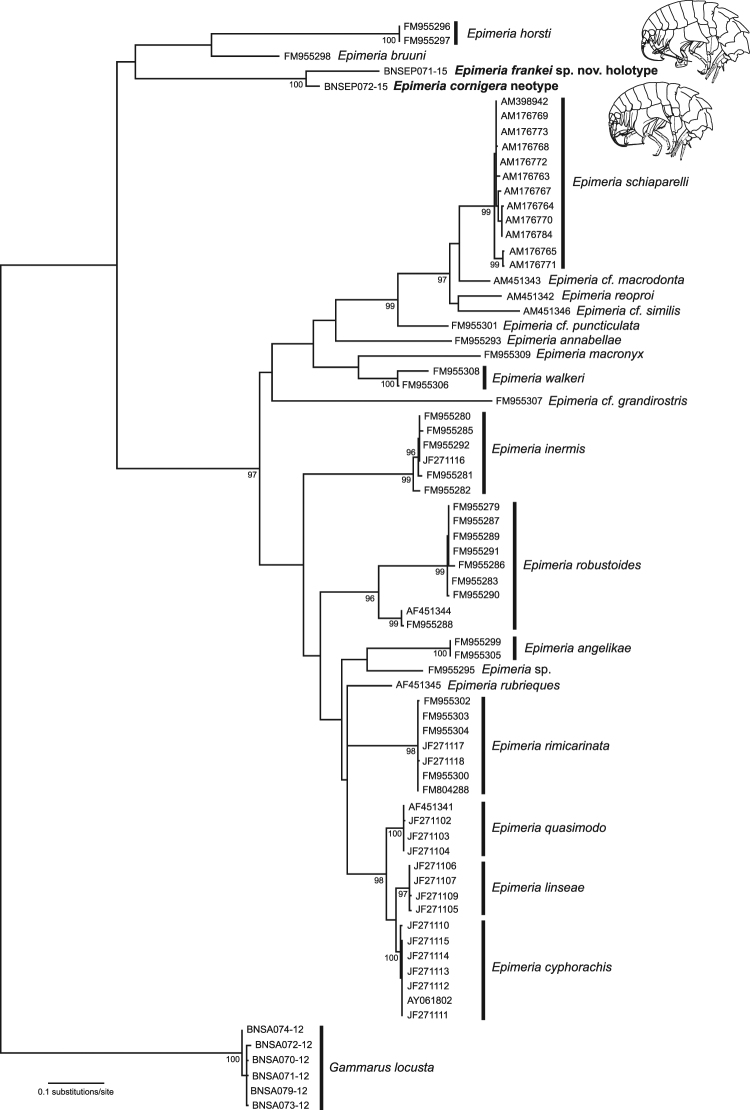
Maximum Likelihood phylogenetic tree of the partial COI sequence data set of 19 *Epimeria* species and *Gammarus locusta* as outgroup. Model of choice: HKY with a log likelihood score of −6124.0843, a discrete gamma distribution (+G, parameter = 1.0064) and a proportion of invariant sites (+I, 46% of sites). Numbers next to internal branches are non-parametric bootstrap values (in %). Only values above 90% are shown. Initial tree(s) for the heuristic search were obtained automatically by applying BioNJ algorithms to a matrix of pairwise distances estimated using the Maximum Composite Likelihood (MCL) approach, and then by selecting the topology with superior log likelihood value. The tree is drawn to scale with branch lengths measured in substitutions per site. All positions with less than 95% site coverage were eliminated. That is, fewer than 5% alignment gaps, missing data, and ambiguous bases were allowed at any position. There were a total of 542 positions in the final dataset.

Our phylogenetic analysis of all available CO1 sequences of *Epimeria* indicated *Epimeria cornigera* as sister taxon *of Epimeria frankei* with a bootstrap support of 100% (Fig. 2). Two species, *Epimeria horsti* Lörz 2008 and *Epimeria bruuni* Barnard 1969, were identified as their sister clade. However, this relationship only had low bootstrap support (40%, not shown). Furthermore, these four *Epimeria* species formed the sister clade of all other analysed *Epimeria* species.

### Small ribosomal subunit (18S rRNA gene)

**Figure 3:**
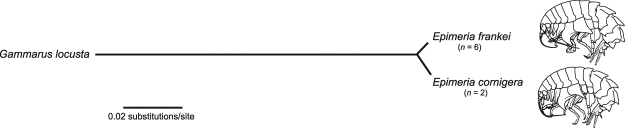
Unrooted Neighbour-joining phylogram of the complete 18S rRNA genes of *Epimeria cornigera*, *Epimeria frankei* sp. nov., and *Gammarus locusta* based on patristic distances.

Eight sequences of the complete 18S rRNA gene were successfully amplified and sequenced for the two species (*Epimeria cornigera*: *n* = 2, *Epimeria frankei*: *n* = 6). We found no insertions or deletions, resulting in a total length of 2,380 bp for both species. As a result of concerted evolution of rRNA gene clusters, we found no intragenomic or intraspecific variations. For all sequences of both species, average base frequencies were A = 0.22, C = 0.23, G = 0.28, and T = 0.27. In total, we observed 34 substitutions and a patristic distance of 2% between both species (Fig. 3; Table S1). Most substitutions (82%) were located in variable regions, with V1 = 1 substitution, V2 = 5 substitutions, V4 = 14, and V7 = 8 (Table S1).

### Mitochondrial genome data: mitochondrial genome reconstruction

The lack of mitogenomic data from closely related species prevented a standard approach of mapping reads to a reference. We therefore implemented independent iterative mapping approaches for both *Epimeria* species. We selected three amphipod bait reference sequences: *Gondogeneia antarctica* (Chevreux, 1906) (Genbank: JN827386.1), *Metacrangonyx goulmimensis* Messouli, Boutin & Coineau, 1991 (Genbank: HE860501.1), and *Onisimus nanseni* (Sars, 1900) (Genbank: NC_013819.1). Regardless of the employed reference, default k-mer values of 31 produced no mappable reads. We therefore carried out the reconstruction using k-mer values of 19 and 25. Both of these values allowed reads to map to our chosen bait reference sequences. However, due to the high level of rearrangements in these distantly related amphipod mitochondria, the correct orientation and therefore continuous sequence of the *Epimeria* mitogenomes could not be recovered using a k-mer value of 19. This k-mer value resulted in multiple mapping initiation points along the reference, leading to a complete, yet fragmented sequence. In contrast, a k-mer value of 25 produced only a single initial mapping point on all three chosen bait reference sequences, allowing a single continuous sequence to be reconstructed and thereby overcoming the above mentioned problems caused by rearrangements.

Regardless of the bait reference sequence used, the final consensus was identical, providing additional reliability to the sequenced genomes. Automated annotations showed the presence of all expected tRNAs, rRNAs and coding genes in an invertebrate mitochondrial genome. Although iterative mapping and consensus sequence reconstruction were performed independently on each species’ genome, annotations showed very similar gene locations and lengths, providing additional support to our approach and final consensus sequences.

**Figure 4:**
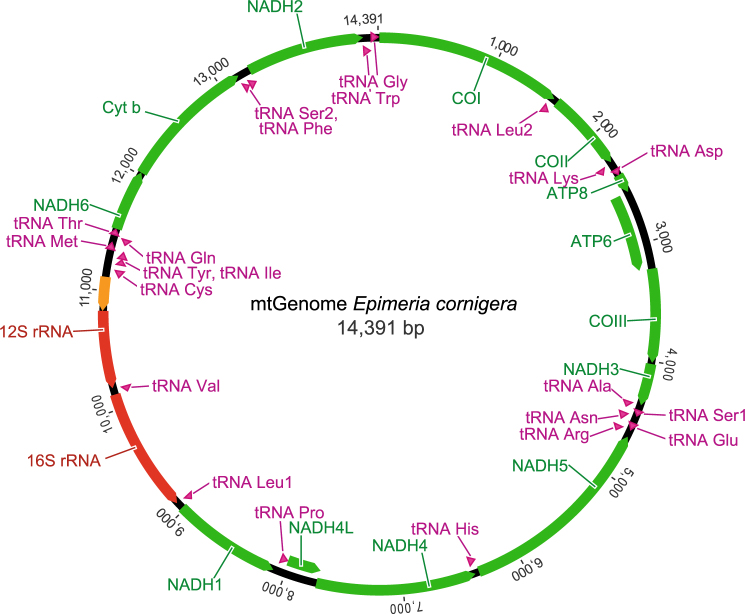
Organization of the mitochondrial genome of *Epimeria cornigera*. Protein encoding genes are marked in green, ribosomal RNAs in red, tRNAs in violet, and the putative control region in orange. Abbreviations: ATP6/8: ATPase subunits 6/8; COI-III: Cytochrome *c* oxidase subunits I–III; NADH2-6/4 L: NADH dehydrogenase subunits 1–6/4 L, and Cyt b: Cytochrome b. All 22 transfer RNA genes are designated by tRNA-X and labeled according to the IUPAC-IUB single-letter amino acid codes. Arrowheads indicate the direction of transcription.

**Figure 5:**
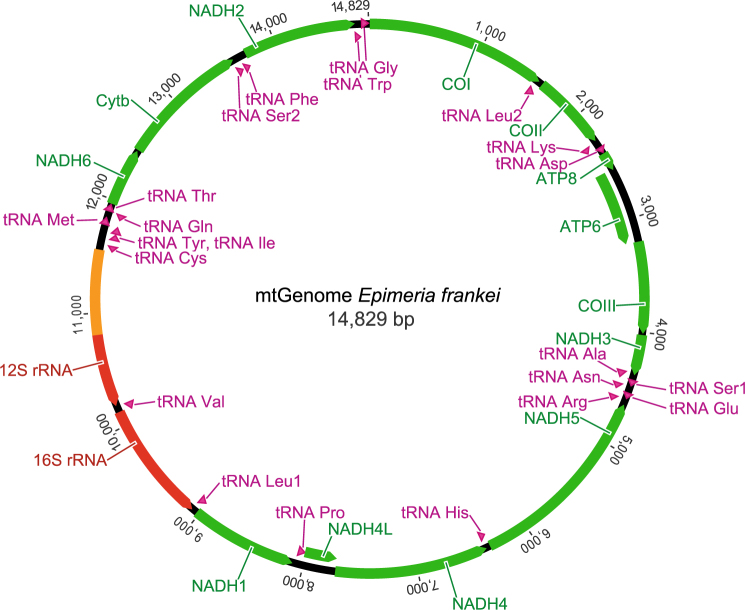
Organization of the mitochondrial genome of *Epimeria frankei* sp. nov. Protein encoding genes are marked in green, ribosomal RNAs in red, tRNAs in violet, and the putative control region in orange. Abbreviations: ATP6/8: ATPase subunits 6/8; COI-III: Cytochrome *c* oxidase subunits I–III; NADH2-6/4 L: NADH dehydrogenase subunits 1–6/4 L, and Cyt b: Cytochrome b. All 22 transfer RNA genes are designated by tRNA-X and labeled according to the IUPAC-IUB single-letter amino acid codes. Arrowheads indicate the direction of transcription.

The complete mitochondrial genomes of *Epimeria cornigera* (14,391 bp) and *Epimeria frankei* (14,829 bp) are typical metazoan mitogenomes. Both consist of a single circular DNA molecule carrying 13 protein-coding genes, two subunits of the mitochondrial ribosomal RNA, 22 tRNAs, one for each amino acid except leucine and serine, which have two copies each, and a putative control region (Figs 4 and 5; Table 2). The heavy strands (H-strand) encode 25 genes (protein coding genes: 9, tRNAs: 16), whereas the remaining 12 genes (protein coding genes: 4, tRNAs: 6, rRNAs: 2) are on the light strands (L-strand). The gene organization of both genomes is similar to the supposed pancrustacean basic pattern^49^ except for the location of various tRNAs and a translocation of a fragment containing two protein-coding genes and one tRNA (NADH6, Cyt *b*, tRNA^Ser2^). There are no gene rearrangements between the two *Epimeria* species investigated in this study. Twenty three gene overlaps and 10 intergenic spacers were found for both mitogenomes. For *Epimeria cornigera*, the total length of overlaps is also 110 bp (ranging from 1 to 26 bp), whereas intergenetic spacers have values from 1 to 44 bp, resulting in a total length of 92 bp, respectively. For *Epimeria frankei*, the total lengths of overlaps are 110 bp, ranging from 1 to 26 bp, and 89 bp (1 to 44 bp) for intergenetic spacers. We found similar nucleotide compositions for both genomes, with frequencies of A = 0.36, C = 0.20, G = 0.10, and T = 0.34 for *Epimeria cornigera* and A = 0.35, C = 0.22, G = 0.10, and T = 0.33 for *Epimeria frankei*.

**Table 1:** K2P distances between *Epimeria cornigera*, *Epimeria frankei* sp. nov., and *Gammarus locusta*.

Species	Mean ISD	Max. ISD	*n*	BIN	Nearest Species	Distance to NN
*Epimeria cornigera* (Fabricius, 1779)	0.14	0.65	23	AAU2062	*Epimeria frankei*	8.69
*Epimeria frankei* sp. nov.	1.06	3.15	57	AAU2061, ACS7228	*Epimeria cornigera*	8.69
*Gammarus locusta* (Linnaeus, 1758)	0.6	1.08	6	AAC0402	*Epimeria frankei*	31.06

Divergence values were calculated for all sequences studied, based on the Nearest Neighbor Summary implemented in the Barcode Gap Analysis tool provided by the Barcode of Life Data System (BOLD). Align sequencing option: BOLD aligner (amino acid based HMM), ambiguous base/gap handling: pairwise deletion. ISD = intraspecific distance. BINs are based on the barcode analysis from 15-02-2017.

**Table 2:** Gene structures of the mitochondrial genomes of *Epimeria cornigera* and *Epimeria frankei* sp. nov.

Gene	Strand	*Epimeria cornigera*					*Epimeria frankei* sp. nov.					*p*-distance
		Start	Stop	Length (bp)	Amino acids	Intergenic^§^	Start	Stop	Length (bp)	Amino acids	Intergenic^§^	
COI	H	1	1563	1563	521	−26	1	1563	1563	521	−26	0.12
tRNA^Leu2^	H	1538	1596	59		0	1538	1596	59		0	
COII	H	1597	2274	678	226	1	1597	2274	678	226	1	0.12
tRNA^Lys^	H	2276	2336	61		−1	2276	2336	61		−1	
tRNA^Asp^	H	2336	2396	61		0	2336	2396	61		0	
ATP8	H	2397	2555	159	53	−7	2397	2555	159	53	−7	0.20
ATP6	H	2549	3220	672	224	−1	2549	3220	672	224	−1	0.14
COIII	H	3220	4008	789	263	9	3220	4008	789	263	9	0.12
NADH3	H	4018	4368	351	117	−3	4018	4368	351	117	−3	0.15
tRNA^Ala^	H	4366	4426	61		−3	4366	4426	61		−3	
tRNA^Ser1^	H	4424	4476	53		−2	4424	4476	53		−2	
tRNA^Asn^	H	4475	4535	61		−4	4475	4535	61		−4	
tRNA^Glu^	H	4532	4594	63		−3	4532	4593	62		−3	
tRNA^Arg^	H	4592	4651	60		−4	4591	4650	60		−4	
NADH5	L	4648	6351	1704	568	−3	4647	6350	1704	568	−3	0.15
tRNA^His^	L	6349	6408	60		−20	6348	6407	60		−20	
NADH4	L	6389	7723	1335	445	−7	6388	7722	1335	445	−7	0.13
NADH4L	L	7717	8013	297	99	2	7716	8012	297	99	2	0.18
tRNA^Pro^	L	8016	8076	61		21	8015	8075	61		21	
NADH1	L	8098	9024	927	309	−4	8097	9023	927	309	−4	0.14
tRNA^Leu1^	L	9021	9080	60		−6	9020	9080	61		−6	
16S rRNA	L	9075	10123	1049		−1	9075	10120	1046		−1	0.08
tRNA^Val^	L	10123	10182	60		−1	10120	10179	60		−1	
12S rRNA	L	10182	10820	639		0	10179	10818	640		0	0.07
ORF	L	10821	11114	294		0	10819	11555	737		0	
tRNA^Cys^	L	11115	11175	61		44	11556	11615	60		44	
tRNA^Tyr^	H	11220	11279	60		2	11660	11719	60		2	
tRNA^Ile^	H	11282	11341	60		−2	11722	11781	60		−2	
tRNA^Met^	H	11340	11402	63		−3	11780	11842	63		−3	
tRNA^Gln^	L	11400	11458	59		−2	11840	11898	59		−2	
tRNA^Thr^	H	11457	11517	61		1	11897	11958	62		1	
NADH6	H	11519	12022	504	168	9	11960	12466	507	169	6	0.15
Cyt*b*	H	12032	13174	1143	381	−5	12473	13615	1143	381	−5	0.15
tRNA^Ser2^	H	13170	13220	51		2	13611	13661	51		2	
tRNA^Phe^	H	13223	13282	60		−1	13664	13723	60		−1	
NADH2	H	13282	14265	984	328	1	13723	14703	981	327	1	0.15
tRNA^Trp^	H	14267	14328	62		−1	14705	14766	62		−1	
tRNA^Gly^	H	14328	14391	64		0	14766	14829	64		0	

The letters H (heavy) or L (light) indicate that the gene is encoded either by the H- or L-strand. ^§^Numbers correspond to the nucleotides separating adjacent genes. Negative numbers indicate overlapping nucleotides.

### Mitochondrial genome data: protein-coding genes

We identified 13 protein-coding genes for both genomes, of which nine were encoded from the H-strand (ATP6, ATP8, COI, COII, COIII, Cyt *b*, NADH2, NADH4, NADH6) and four from the L-strand (NADH1, NADH4, NADH4L, NADH5) (Figs 4 and 5; Table 2). All proteins have typical invertebrate mitochondrial start codons (ATA, ATC, ATG, ATT, or TTG) and stop codons (TAA, TAG). Whereas 11 protein-coding genes have identical numbers of base pairs within both genomes, different lengths were found for two genes: NADH2 (*Epimeria cornigera*: 984 bp, 328 amino acids; *Epimeria frankei*: 981 bp, 327 amino acids) and NADH6 (*Epimeria cornigera*: 504 bp, 168 amino acids; *Epimeria frankei*: 507 bp, 169 amino acids). Nucleotide divergence (patristic distances) between the protein-coding genes of both mitogenomes ranged from a minimum of 0.12% for COI, COII and COIII to a maximum of 0.20% for ATP8 (Table 2).

### Mitochondrial genome data: ribosomal and transfer RNA genes

A total of 22 tRNA genes with lengths from 51 bp (tRNA^Ser2^) to 64 bp (tRNA^Gly^) were found within both mitochondrial genomes (Figs 4 and 5; Table 2). The data revealed differences in lengths (+/−1 bp) for four tRNAs: tRNA^Glu^, tRNA^Leu1^, tRNA^Cys^, and tRNA^Thr^. Sixteen tRNAs are encoded on the H-stand whereas six tRNAs are located on the L-strand. All tRNAs are capable of folding into a typically clover-leaf-like secondary structure except tRNA^Ser1^ and tRNA^Ser2^. As previously reported for other crustacean taxa, these two tRNAs cannot form a secondary structure due to their lack of the dihydrouracil arms^50,51^. Both rRNA genes were found on the L-strand. They are located between the tRNA^Leu1^ and the putative control region and are separated by tRNA^Val^. Gene lengths of the 12S rRNA genes are 639 bp for *Epimeria cornigera* and 640 bp for *Epimeria frankei*, whereas the lengths of 16S rRNA genes are 1,049 bp (*Epimeria cornigera*) and 1,046 bp (*Epimeria frankei*). The nucleotide divergence (patristic distance) was 0.07% between the 12S rRNA genes and 0.08% between the 16S rRNA genes (Table 2).

### Mitochondrial genome data: non-coding regions

The major non-coding regions (*Epimeria cornigera*: 294 bp, *Epimeria frankei*: 737 bp) were identified between the 12S rRNA and tRNA^Cys^ genes, which are considered to represent the putative control regions (Table 2). It should be noted that the read depth in this region was relatively high in *Epimeria cornigera* compared to the rest of the genome. This could indicate the presence of a repetitive element that could not be be properly assembled, All other 10 non-coding regions are smaller, ranging from 1 to 44 bp for both genomes. The A+T contents of the control regions are higher than those of other mitogenome regions, with 0.78 for *Epimeria cornigera* and 0.81 for *Epimeria frankei*. Furthermore, both control regions consist of several repetitive motifs in tandem that are different among both genomes.

### Morphological distinction

**Figure 6:**
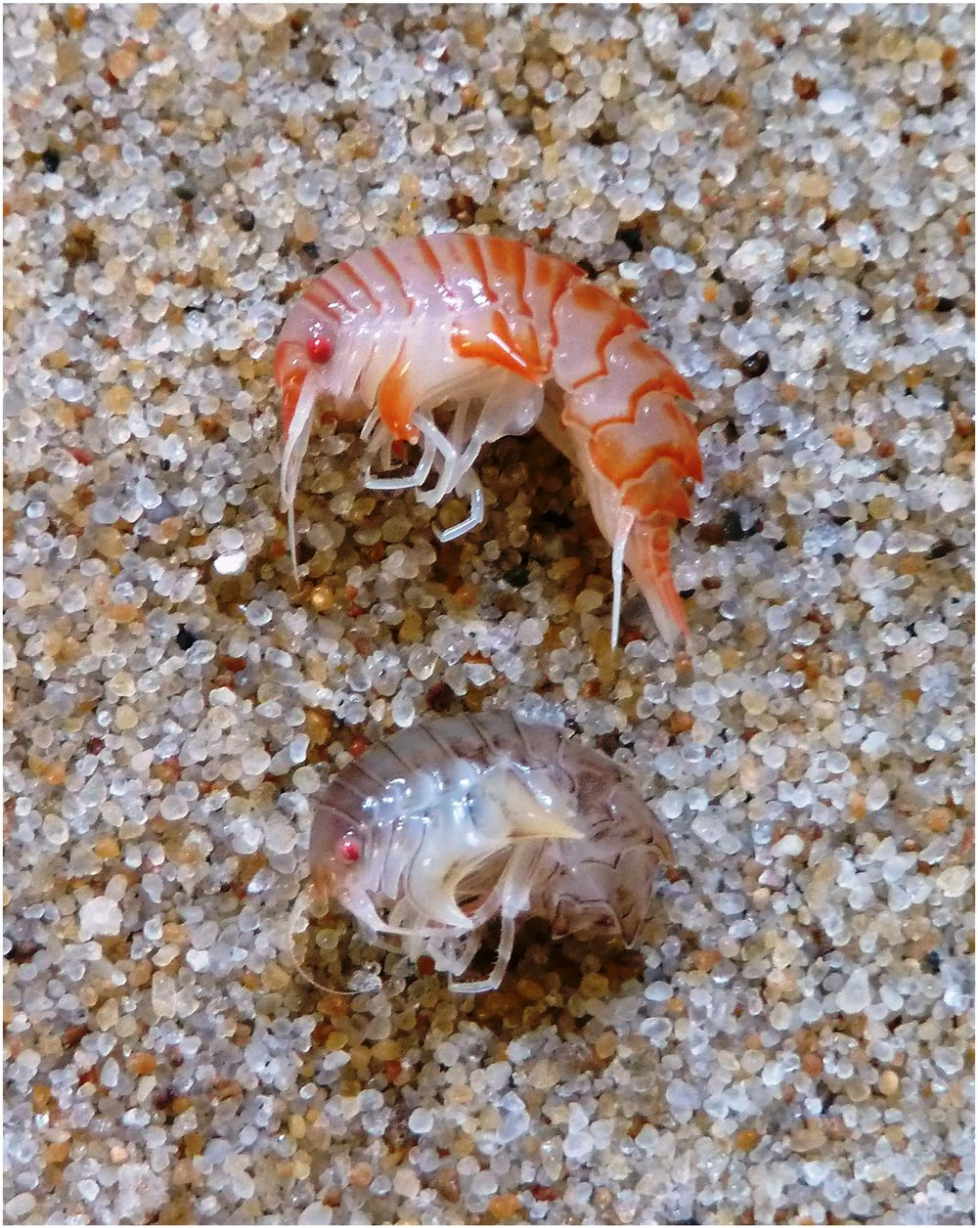
Photograph of freshly caught individuals of *E*. *cornigera* (top) and *E*. *frankei* sp. nov. (bottom); body colouration can clearly be used to discriminate the two species.

*Epimeria frankei* can be distinguished from *E*. *cornigera* by the shape of coxal plate 5. In *E*. *frankei*, the anteroventral margin of coxa 5 describes a nearly straight line or is only slightly concave, whereas this margin is strongly concave in *E*. *cornigera* (less pronounced in males). Coxa 5 is strongly extended posteroventrally in both species, coming along with an only slightly concave posterior margin in *E*. *cornigera* (distinctly concave in *E*. *frankei*). Additionally, urosomite 3 can be used as a diagnostic character. In *E*. *frankei*, urosomite 3 is pronounced in a distinctly upwards pointed projection (Fig. 8D), whereas urosomite 3 of *E*. *cornigera* is characterised by an inconspicuous blunt hump (Fig. 14D) or is not pronounced at all. The most conspicuous difference between the two species, however, can be observed on live or freshly caught individuals (observed on approx. 100 indiviuals of each species): *E*. *frankei* features an even, brownish or grey colouration with hints of purple or even pink (Fig. 6). In contrast, *E*. *cornigera* is characterised by an eye-catching red-white colouration (Fig. 6). Unfortunately, the colouration of individuals vanishes quickly when being exposed to ethanol, resulting in yellowish or pale white specimens in both species.

## Discussion

The current study fully implements the concept of integrative taxonomy, proposed as a framework to bring together conceptual and methodological developments, combining as many different data types as possible to characterise species in a multifaceted and detailed way^1,52,53^. Here, two sibling amphipod species are described using comprehensive morphological, genetic, and mitogenomic data. This is the first use of genomic data (here: complete mitochondrial genomes) as part of a species description and a redescription within the Metazoa, setting a new desirable standard for future species descriptions^35^. We are convinced that modern species descriptions should seek for this integrative addition of comprehensive complementary information, ensuring the highest level of confidence for both biodiversity and ecological research.

### Biology and geographic distributions

Species of the genus *Epimeria* are morphologically characterised by dorsal teeth and/or robustly elongated coxal plates 1–4. The coxal plates exhibit an ‘inter-locking’ mechanism which may have a function during the mating or as defence mechanism against predators^54^. A number of *Epimeria* species are frequently recorded from shallow to deep waters of the northern Atlantic and Arctic waters. Most *Epimeria* species are bottom-dwellers and were often assumed to be associated with other biota such as deep-sea corals, hydrozoans, sponges and holothurians in the North Atlantic^55–57^, but reliable information on their life styles is scarce. With the description of *E*. *frankei*, the number of species in the north-eastern Atlantic increases to a total of 5: *E*. *cornigera* (current study), *E*. *frankei* (sp. nov.; current study), *E*. *loricata* G.O. Sars 1879, *E*. *parasitica* M. Sars 1858 and *E*. *tuberculata* G.O. Sars 1893. The main morphological characteristics that can be used for their distinction are provided in the key below.

**Figure 7:**
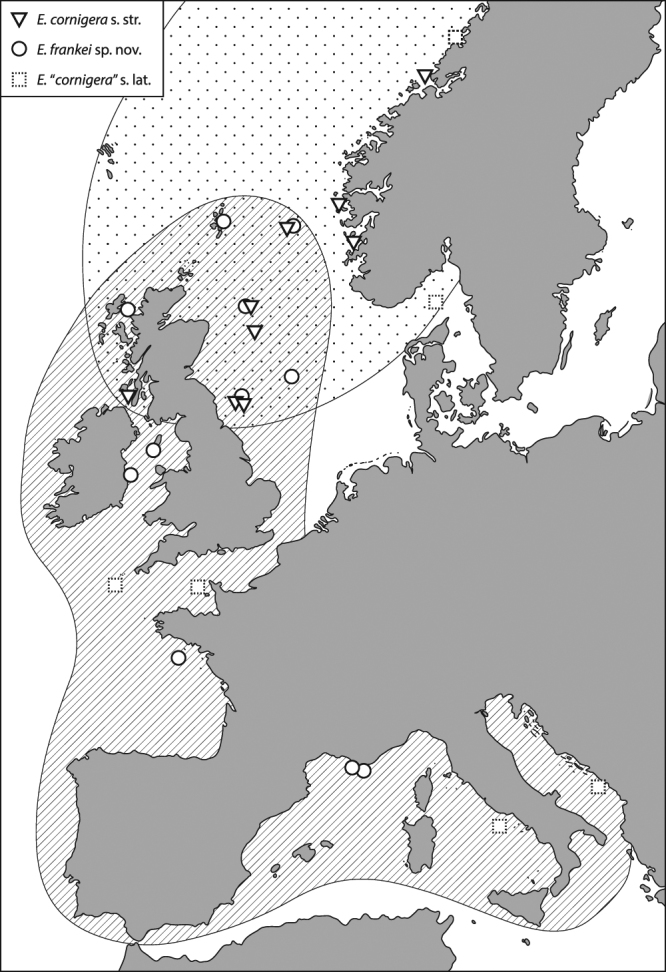
Suggested geographic distributions of *E*. *cornigera* and *E*. *frankei* sp. nov. considering the examined material of the current study and previous records of “*Epimeria cornigera* s. lat.” (i.e. published records of “*E*. *cornigera*” that cannot be assigned clearly) in the literature^56,109,110,114,120–123^. Initial map built with ArcGIS 10.3.1 (www.esri.com/arcgis) and modified with Adobe Illustrator (CS 6).

Former reports of *E*. *cornigera* s. lat. ranged from the NE Atlantic to the Mediterranean Sea. In fact, the current findings and the geographic information of the re-examined museum material suggest that *E*. *cornigera* s. str. may be restricted to the northern East Atlantic, whereas *E*. *frankei* seemingly exhibits a Mediterranean-Lusitanean distribution (Fig. 7). However, as also evidenced by the same type locality of the two species, both occur sympatrically on sandy bottoms of the North Sea and near the northern British coasts where water temperatures are probably suitable for both species.

#### Key to the Epimeria species of the north-eastern Atlantic

1. All pereonites dorsally carinate.... *E*. *loricata*
  Only pereonites (5) 6–7 dorsally carinate.... **2**
2. Apical tips of coxae 1–5 bluntly rounded; coxa 4 projecting ventrally with concave posteroapical margin; coxa 5 moderately extended posteroventrally (about as long as broad).... *E*. *tuberculata*
  Apical tips of coxae 1–5 rounded or subacute; coxa 4 strongly projecting ventrally and posteroapical margin strongly concave; coxa 5 strongly extended posteroventrally (at least twice as long as broad).... **3**
3. Epimeral plate 3 angular, produced to a large posterodistal projection, posterior margin straight.... *E*. *parasitica*
  Epimeral plate 3 biangular with subacute posterodistal tooth.... **4**
4. Coxa 5 anteroventral margin nearly straight or only slightly concave; urosomite 3 with pointed projection; body colouration of live animals single-coloured brown to grey with hints of purple.... *E*. *frankei* sp. nov.
  Coxa 5 anteroventral margin strongly concave; urosomite 3 not produced or only with blunt hump; body colouration of live animals white with red stripes.... *E*. *cornigera*

### DNA barcodes

During the last few years it has become evident that DNA barcodes represent powerful diagnostic supplementary characters which accelerate and revive traditional morphological taxonomy by providing additional data^58^. Consequently, there is continuous progress of using DNA barcodes as part of species descriptions in the Crustacea^24^. The analysis of our obtained DNA barcode data revealed distinct clusters for both species with a minimum interspecific distance of 8.69% (K2P) (Table 1). This value is similar to interspecific distances known from other closely related *Epimeria* species, with minimum distances of 8.5%^59^. Furthermore, we found two lineages and two BINs (AAU2061, ACS7228) for *Epimeria frankei* (Fig. 1). However, as nuclear 18S rRNA gene sequences from specimens of both lineages were identical and no corresponding morphological differences were observed, these have to be considered as one species unless further data becomes available (Fig. 3). Therefore, the observed variability may be the result of phylogeographic effects in *E*. *frankei*^60–62^.

### Phylogenetic analysis of the CO1 sequences

About 66% of the currently known species of *Epimeria* are found around Antarctica as part of a monophyletic lineage^59,63–65^, representing the best known example of a species flock within Antarctic amphipods^66^. This hypothesis is supported by our phylogenetic results: all studied species from Antarctica form a monophyletic clade with high bootstrap support (97%) (Fig. 2). Our analysis indicates a potential relationship of *Epimeria cornigera* and *Epimeria frankei* with two non-Antarctic species: *Epimeria horsti* and *Epimeria bruuni*. Both species are only known from waters around New Zealand so far^67^. However, this relationship is supported only by a low bootstrap value (40%). A more comprehensive analysis based on i) further, particularly non-Antarctic species, ii) more molecular data (both mitochondrial and nuclear) and iii) morphological characteristics is needed to reconstruct the phylogeny of this impressive amphipod genus in detail.

### Mitochondrial genomes

Because modern high-throughput sequencing technologies allow for much faster and easier analyses of genomes, the analysis of mitogenomes will likely increase dramatically in the near future^35^. Compared to other crustacean groups, the number of available complete mitochondrial genomes for amphipods is quite high (*n* = 29, 2017-06-21) and these display various patterns of gene arrangements^68^. Mitogenomes of both *Epimeria cornigera* and *Epimeria frankei* represent typical circular metazoan mitogenomes with an identical gene arrangement (Table 2) and a patristic distance of 0.12% between both genomes. Patristic distances between all protein-coding genes range from 0.12% (COI, COII) to 0.20% (ATP6), and in the case of rRNA genes from 0.07 (12 rRNA) to 0.08 (16S rRNA). With a length of 737 bp the putative control region of *Epimeria frankei* is significantly longer than in *Epimeria cornigera* (294 bp). This region is the largest non-coding part of the mitochondrial DNA in general and includes the major regulatory elements for its replication and expression^69^. Furthermore, it is the most polymorphic part of the mitochondrial genome with hypervariable sites^70^. Nevertheless, we found two conserved parts shared between both genomes, covering 80 and 190 bp, respectively. Our results support the concept that the control region is divided into three polymorphic domains, being separated by two stretches with no interspecific variability^71^. However, more data have to be evaluated to analyse this phenomenon in more detail.

## Material and Methods

### Sample collection and DNA extraction

In total, 75 specimens were collected with beam trawls in the North Sea between 2012 and 2014 as part of the German Small-scale Bottom Trawl Survey (GSBTS^72^). The amphipods were preserved in ethanol (96%) directly after collection. All specimens were morphologically identified by JB and HN. For each specimen, total genomic DNA was extracted from one to three dissected pereopods using the QIAmp^©^ Tissue Kit (Qiagen GmbH, Hilden, Germany) following the supplier’s extraction protocol.

Specimens were deposited in the collections of natural history museums (see below), whereas DNA extracts were listed and stored in the North Sea Fauna collection of the German Centre for Marine Biodiversity Research (DZMB) in Wilhelmshaven, Germany.

### Amplification and sequencing: DNA barcodes and complete 18S rRNA gene sequences

Polymerase chain reaction (PCR) was used to amplify the DNA barcode fragment using the primer pair LCO1490 and HCO2198^19^ or the newly designed degenerated amphipod-specific primer pair CO1-LCO-AMP1 (5′-THT CDA CHA ACC AYA AAG AYA TYG G-3′) and CO1-HCO-AMP2 (5′-ATD ACT TCW GGR TGV CCR AAR AAY C-3′). For the latter, we added derived M13 forward and reverse tails to both primers to provide known primer-binding sites for sequencing^73^. Therefore, the complete primer sequences were 5′-TGT AAA ACG ACG GCC AGT THT CDA CHA ACC AYA AAG AYA TYG G -3′ for CO1-LCO-AMP1 and 5′-CAG GAA ACA GCT ATG AC ATD ACT TCW GGR TGV CCR AAR AAY C-3′ for CO1-HCO-AMP2 (with underlined M13 tail sequences). Amplification reactions were carried out on a Mastercycler pro S system (Eppendorf, Hamburg, Germany), using illustra^TM^ puReTaq Ready-To-Go PCR Beads (GE Healthcare, Buckinghamshire, UK) in a total volume of 20 µl, containing 17.5 µl sterile molecular grade H_2_O, 2 µl DNA template with an DNA amount between 2 and 150 ng/µl and 0.25 µl of each primer (20 pmol/µl) for amplification. For this gene fragment (CO1), PCR thermal conditions included an initial denaturation at 94 °C (5 min), followed by 38 cycles at 94 °C (denaturation, 45 s), 48 °C (annealing, 45 s), 72 °C (extension, 80 s), and a final extension step at 72 °C (7 min).

Furthermore, the complete nuclear 18S rRNA gene was amplified for selected individuals of both species (*Epimeria cornigera*: *n* = 2, *Epimeria frankei*: *n* = 6). Detailed information about used primer pairs, amplification reactions and temperature profiles are provided in previous studies^74,75^. For verification, negative and positive controls were included with each round of reactions. Three μl of the amplified products were inspected for size conformity by electrophoresis in a 1.5% agarose gel with GelRed^TM^ using commercial DNA size standards. The remaining PCR product was purified with the QIAquick^©^ PCR Purification Kit (Qiagen GmbH, Hilden, Germany). Purified PCR products were cycle sequenced and sequenced in both directions at a contract sequencing facility (GATC, Konstanz, Germany) using the M13 sequence tails for the DNA barcodes or the same primers as employed in the PCR in the case of the 18S rRNA gene. For this marker, some additional internal primers were used for sequencing: Forward: AF700F_mod, 1000 F, 1250FN_mod; Reverse: 700 R, 1155 R, 1500R_mod^75,76^. Complementary sequences were assembled with the Geneious version 9.0.4 program package^77^. BLAST searches were performed to confirm the identity of all new sequences^78,79^. Furthermore, Geneious was used to translate all COI sequences to amino acid sequences to check for presence of nuclear mitochondrial pseudogenes (numts).

All relevant voucher information, photos, DNA barcodes, used primer pairs and trace files are publicly accessible through the public data set “North Sea Epimera” (Dataset ID: DS-EPNS Epimeria North Sea; dx.doi.org/10.5883/DS-CRNS) on the Barcode of Life Data Systems (BOLD; www.boldsystems.org)^80,81^. All new sequence data were deposited in GenBank (CO1: MG191712-MG191786, 18S: MG191704-MG191711).

### Mitochondrial genome: library building, sequencing and raw read processing

DNA extracts were built into double-barcoded Illumina sequencing libraries using a published protocol set^82^, with some minor modifications^83^. The optimal number of cycles for amplification was established using qPCR to avoid saturation. Sequencing was performed on the Illumina Nextseq. 500 at the University of Potsdam, Germany, using 150 base pair (bp) paired-end (PE) reads. 25,368,699 PE reads were sequenced for the holotype of *Epimeria frankei* and 20,329,986 for the neotype of *Epimeria cornigera*. Illumina adapter sequences were trimmed from read ends, and all reads less than 30 bp were discarded using Cutadapt 1.4^84^. PCR duplicate sequences were removed using Fastuniq^85^. Forward and reverse sequences were interleaved into a single file using a perl script available in the MITObim package^86^.

### Mitochondrial genome: sequence reconstruction

We carried out independent mitochondrial reconstructions for both species using MiTObim v1.8^86^, an iterative mapping wrapper script for the Mira v4.0.2 assembler^87^. We implemented a direct reconstruction without prior mapping assembly using default parameters with alterations to mismatch values, *k*-mers and reference bait sequences. The utilised bait sequences consisted of: *Gondogeneia antarctica* (JN827386.1), *Metacrangonyx goulmimensis* (HE860501.1), and *Onisimus nanseni* (NC_013819.1). The interleaved fastq files were treated as single end data. Reconstructions were conducted using *k*-mer values of 19, 25 and 31. We implemented a reconstruction protocol based on that found in Westbury *et al*.^88^ by beginning with the most strict mismatch value (0%) and gradually increasing this value until the mitochondrial genome was complete. Mismatch values of 0–8% were implemented for *Onisimus nanseni* and *Metacrangonyx goulmimensis* whereas mismatch values of 0–6% were implemented for *Gondogeneia antarctica*. This resulted in 25 separate iterative mapping alignments.

Mira output maf files were converted to sam files and visualised using Geneious. For each independent iterative mapping alignment, a 20x minimum coverage was implemented for consensus building along with a strict 95% threshold for base calling. This resulted in 25 individual consensus sequences, nine constructed using *Onisimus nanseni* as reference, nine using *Metacrangonyx goulmimensis* as reference and seven from using *Gondogeneia antarctica* as reference. From this data, we constructed three reference-specific consensuses by aligning all consensus sequences produced via mapping to the same reference bait sequence, i.e. consensuses produced from different mismatch values but the same reference sequence, using Mafft^89^ and a 50% strict consensus base call. These three reference-specific consensus sequences were then aligned in Mafft and a final consensus was built using a 50% strict consensus base call. We performed automatic annotations with MITOS^90^ to check for the presence of genes and tRNAs in our final consensus sequence. The tRNAs were also annotated with ARWEN v1.2^91^. Finally, gene limits were refined by comparison with orthologous mtDNA sequences of other amphipod genomes. Both new genomes were deposited in GenBank (accession numbers: *Epimeria cornigera*: MF361127, *Epimeria frankei*: MF361126).

## Sequence analysis

### DNA barcodes

In addition to the 75 CO1 sequences of both species obtained for this study, five already published sequences of *Epimeria cornigera* (accession numbers: KT208490, KT208918, KT209458, KT209498, KT209569) and six barcode sequences of the outgroup *Gammarus locusta* (Linnaeus, 1758) (KT208462, KT208572, KT208951, KT209189, KT209193, KT209211) were retrieved from GenBank^28^. Thus, the complete data set consisted of 86 DNA barcodes.

Intra- and interspecific nucleotide distances of the analysed amphipods were based on the Kimura 2-parameter model (K2P)^92^, using the analytical tools on the BOLD workbench (align sequences: BOLD aligner; ambiguous base/gap handling: pairwise deletion). We also used BOLD to calculate base frequencies of all barcodes. All barcode sequences were assigned to the Barcode Index Number (BIN) system which is implemented in BOLD^81^. All sequences were aligned using MUSCLE version 3.6^93^ with default settings. In a further step, a neighbor-joining cluster analysis (NJ)^94^ was performed to reconstruct a phylogenetic tree based on K2P distances for all DNA barcode sequences using MEGA7.0.18^95^. Non-parametric bootstrap support values were obtained by resampling and analysing 1,000 replicates^96^.

In order to explore the phylogenetic position of *Epimeria cornigera* and *Epimeria frankei*, we reconstructed the phylogeny of the genus *Epimeria* in a maximum-likelihood framework using MEGA7.0.18. For the analysis, COI barcodes of the neotype of *Epimeria cornigera* and the holotype of *Epimeria frankei* were used as well as all available COI sequences of the DNA barcode fragment from NCBI GenBank with a length of at least 500 base pairs (*n* = 67, date of download: 01-02-2017), representing 16 of the 84 currently recognized species in the genus *Epimeria* (29%). A table of all included species and corresponding accession numbers is included in the supplementary information (Table S2). All sequences were aligned using MUSCLE version 3.6^93^ with default settings and with the six sequences of *Gammarus locusta* as outgroup. The most appropriate model of sequence evolution was determined beforehand using the Decision Theory performance-based selection (DT) as implemented in jModeltest 2.1.1^97^. The Hasegawa-Kishino-Yano model (HKY85)^98^ was shown to be the optimal nucleotide substitution model with the following parameters: nucleotide frequencies A: 0.32, C: 0.24, G: 0.15, T: 0.29; transition/transversion ratio = 3.4401; gamma distribution shape = 0.885; and proportion of invariable sites = 0.439. Node support for individual clades was estimated using 1,000 non-parametric bootstrap replicates^96^.

### Small ribosomal subunit (18S rRNA)

All eight 18S rDNA sequences of the two *Epimeria* species and one outgroup sequence of *Gammarus locusta* (AF419222) were aligned with MUSCLE version 3.6 using default settings. The software package MEGA7.0.18 was used to calculate nucleotide frequencies, interspecific distances based on patristic distances and to perform a NJ cluster analysis for a graphical representation of nucleotide divergence. Secondary structure elements were identified using the CRW site^99^ and other published models^100–102^.

### Mitochondrial genomes

An initial alignment of both genomes was obtained with Clustal W^103^ as implemented in BioEdit v7.2.6^104^. Start and end positions of all encoding genes were investigated using the Geneious software package. The nucleotide composition of both mitogenomes and patristic distances of all mitochondrial proteins were calculated with MEGA7.0.18. A graphical representation of both genomes was produced with the Geneious package.

### Morphological analyses

Before examination, specimens were transferred to 70% ethanol and appendages were temporarily mounted in glycerin. Whole animals and appendages were then studied and drawn under a Leica M205c microscope equipped with a drawing tube. All material was later conserved in ethanol (95%). Pencil drawings were scanned and inked with the software Adobe Illustrator (CS6) on Wacom drawing tablets (Intuos 5, A4 and A3), following the methods described by Coleman^105,106^. Referring to the considerations of d’Udekem d’Acoz^107^ and Krapp-Schickel^108^, the following terminology was applied in the descriptions: seta = slender, flexible articulated structure; spine = robust, inflexible articulated structure (synonymous to ‘robust seta’); tooth = non-articulated, pointed ectodermal structure. Although officially listed in the collections of the Natural History Museum of Denmark, Copenhagen, Fabricius’ holotype is missing (pers. comm. Jørgen Olesen) and was thus presumed as lost in the current study. Furthermore, there is no exact information on the type locality, which can only be assumed to be located in the northern North Sea. Therefore, types were chosen considering the geographical area where both species were commonly found sympatrically. This was further validated by the descriptions and drawings of Sars^55^, which seem to refer to *E*. *cornigera s*. *str*. All type material was deposited in the Natural History Museum, London, UK (NHMUK). Further specimens from the current study were transferred to the natural history museums of Berlin and Frankfurt including their original accession numbers of the former collection of the Molecular Taxonomy (MT) group at DZMB. Additional material was examined in the collections of the NHMUK and the Museo Civico di Storia Naturale, Verona, Italy (MCSN).

## Systematics

**Figure 8:**
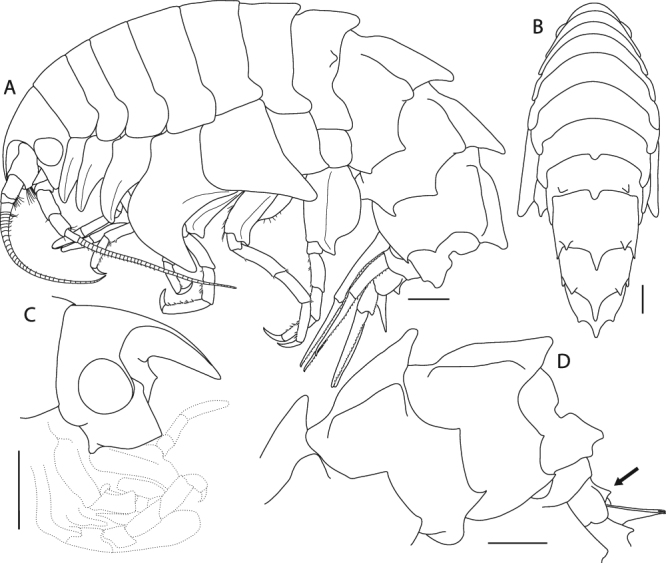
*Epimeria frankei* sp. nov., holotype, adult female, northern North Sea, NHMUK 2015.3264: (**A**) lateral habitus, (**B**) dorsal habitus, (**C**) head in lateral view, (**D**) pleon and urosome; all scales 1 mm.

**Figure 9:**
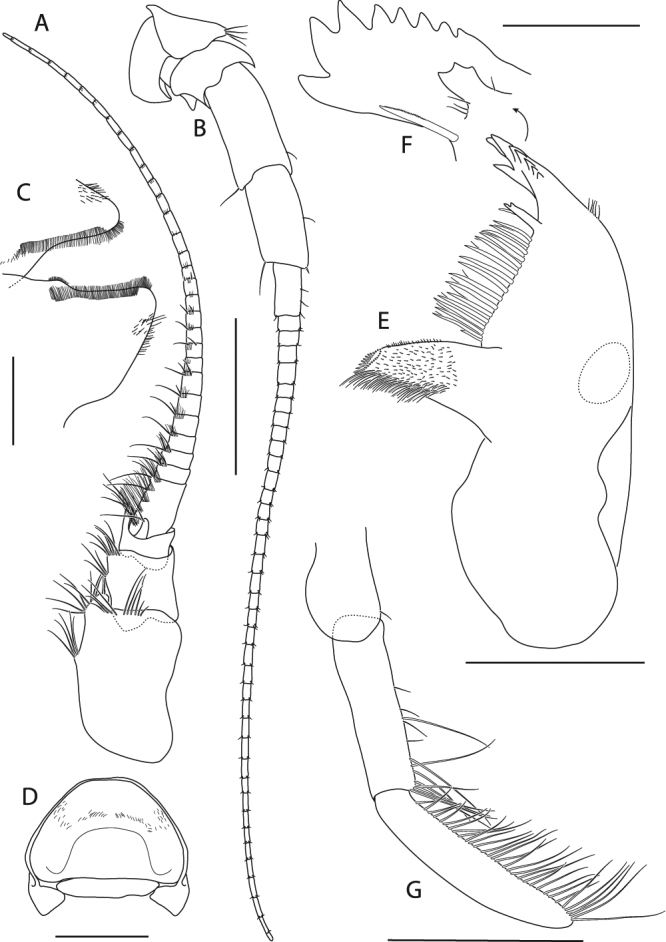
*Epimeria frankei* sp. nov., holotype, adult female, northern North Sea, NHMUK 2015.3264: (**A**) antenna 1, (**B**) antenna 2, (**C**) hypopharynx, (**D**) mandible, (**E**) mandible incisor and lacina mobilis, (**F**) mandibular palpus, (**G**) labrum; scales: (A,B) 1 mm; (C,D,E,G) 0.5 mm; (F) 0.25 mm.

**Figure 10:**
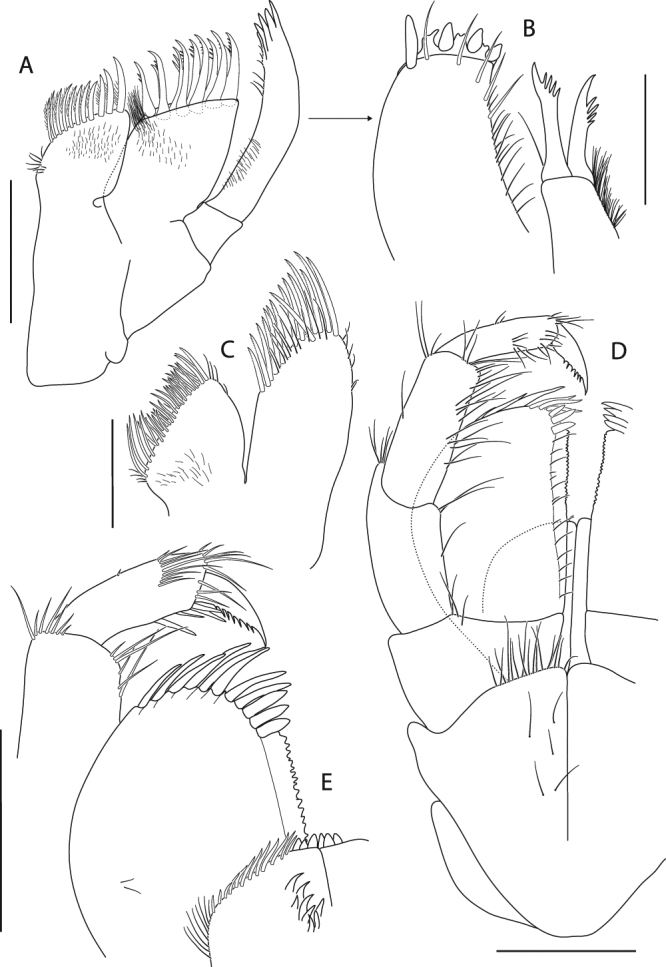
*Epimeria frankei* sp. nov., holotype, adult female, northern North Sea, NHMUK 2015.3264: (**A**) maxilla 1, (**B**) maxilla 1 palpus and outer plate distal ends lateral view, (**C**) maxilla 2, (**D**) maxilliped, (**E**) maxilliped palpus and outer plate; scales: (A,D,E) 0.5 mm; (B,C) 0.25 mm.

**Figure 11:**
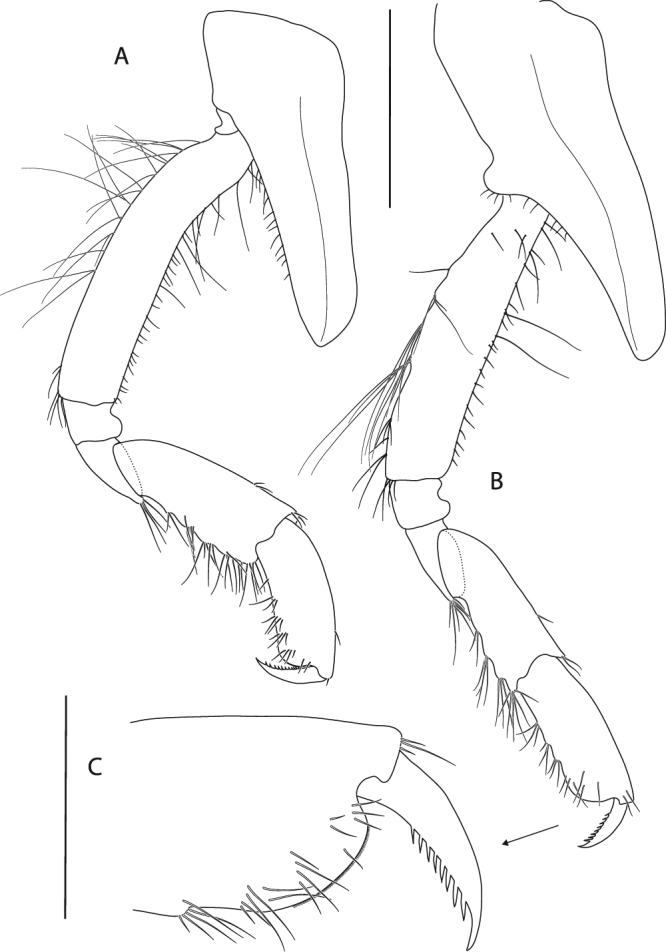
*Epimeria frankei* sp. nov., holotype, adult female, northern North Sea, NHMUK 2015.3264: (**A**) gnathopod 1, (**B**) gnathopod 2, (**C**) distal articles of gnathopod 2; scales: (A,B) 1 mm; (C) 0.3 mm.

**Figure 12:**
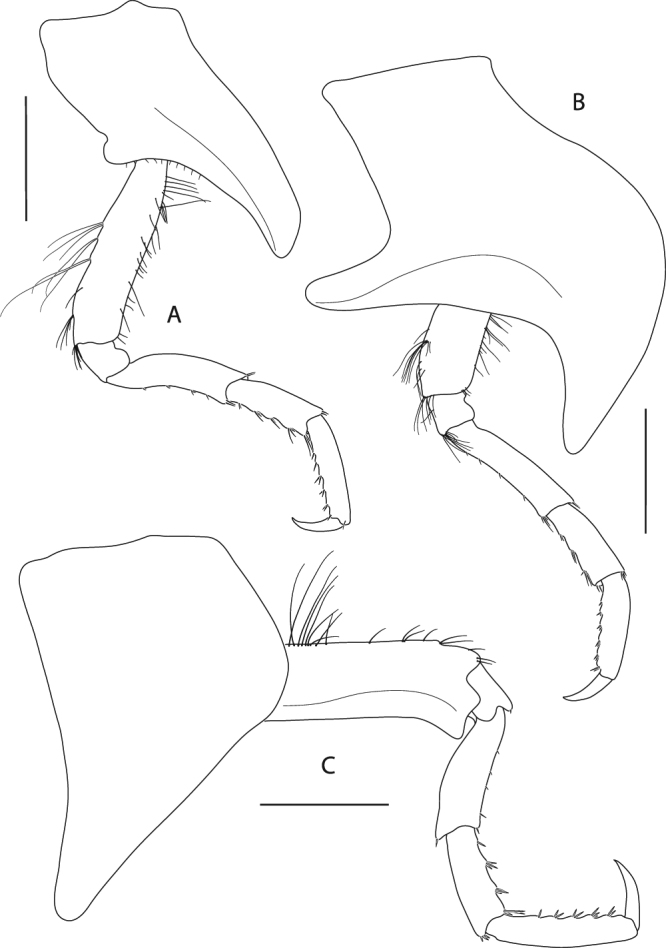
*Epimeria frankei* sp. nov., holotype, adult female, northern North Sea, NHMUK 2015.3264: (**A**) pereopod 3, (**B**) pereopod 4, (**C**) pereopod 5; all scales 1 mm.

**Figure 13:**
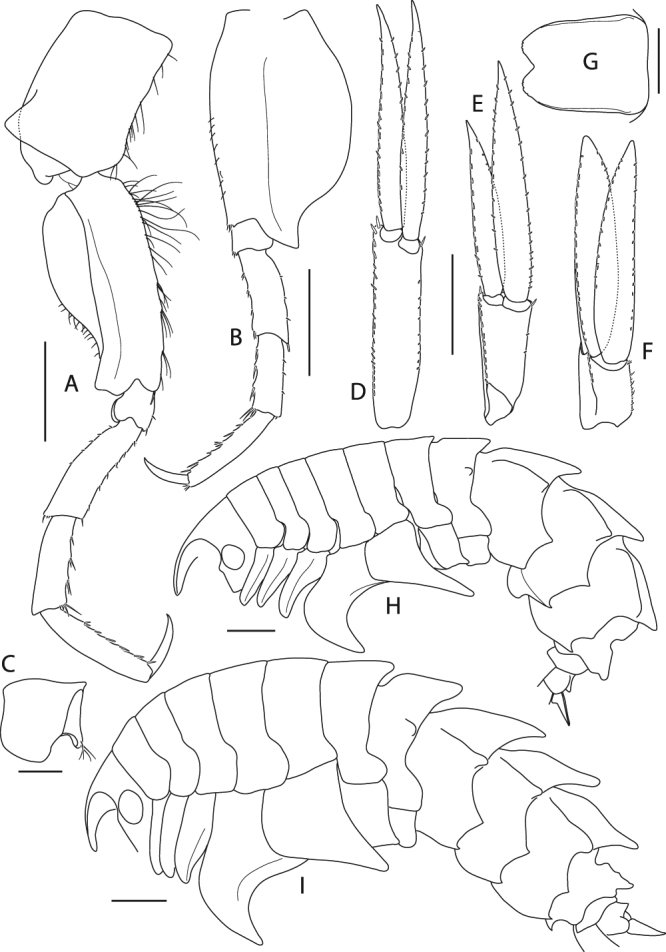
*Epimeria frankei* sp. nov., holotype, adult female, northern North Sea, NHMUK 2015.3264: (**A**) pereopod 6, (**B**) left pereopod 7, (**C**) right coxa 7, (**D**) uropod 1, (**E**) uropod 2, (**F**) uropod 3, (**G**) telson; *Epimeria frankei* sp. nov., paratype, adult male, northern North Sea, NHMUK 2015.3267: (**H**) lateral habitus; *Epimeria frankei* sp. nov., adult female, Mediterranean Sea, MCSN Ec1: (**I**) lateral habitus; scales: (A,B,D,E,F,H,I) 1 mm; (C,G) 0.5 mm.

***Epimeria frankei*** **sp**. **nov**. **Beermann & Raupach** (Figs 8, 9, 10, 11, 12 and 13).

*Epimeria cornigera* Chevreux & Fage^109^, 1925: 191, fig. 198–200. – Karaman^110^, 1972: 143. – Ledoyer^111,112^, 1977: 397; 1993: 616, fig. 423.

### Material examined

Holotype (NHMUK 2015.3264), ovigerous female, 19 mm, northern North Sea – approx. 150 km west of Bergen (60.3104°, 2.4967°), in 97 m water depth, 05.viii.2013, coll. H. Neumann. Paratypes: two ovigerous females (NHMUK 2015.3265 - 3266), locality data identical to holotype; one adult male (Fig. 13; 13 mm; NHMUK 2015.3267), northern North Sea (60.3956°, 2.5788°), 86 m, 30.vii.2012, coll. H. Neumann.

### Other material

One ovigerous female (MT07112), North Sea (57.9514°, −0.7692°), 101 m, 05.viii.2012, coll. H. Neumann. Fourteen males and 14 females (of which 7 ovigerous, 6 juvenile; MT06718, MT06742-6749, MT06755-6764, MT07102-7110), western North Sea (54.8732°, −0.9581°), 68 m, 07.iii.2012, coll. H. Neumann. Nine males (1 juvenile) and eight females (4 ovigerous, 4 juvenile; MT06787-7101, MT06712-6713), central North Sea (56.093°, 1.213°), 87 m, 06.iii.2012, coll. H. Neumann. One adult female (MT06708), northern North Sea (60.3659°, 2.4386°), 96 m, 31.vii.2012, coll. I. Rottgardt. Two adult females (1 ovigerous; MT06706-6707), northern North Sea (60.3583°, 2.5371°), 106 m, 31.vii. – 01.viii.2012, coll. I. Rottgardt. One juvenile female (MT06704), northern North Sea (60.3459°, 2.6711°), 101 m, 30.vii.2012, coll. H. Neumann. One juvenile female (MT06703), western North Sea (55.1097°, −0.5023°), 82 m, 07.ii.2012, coll. H. Neumann. One juvenile female (MT06701), western North Sea (55.4147°, −0.0218°), 75 m, 07.iii.2012, coll. H. Neumann. One male (?) and three ovigerous females (MT01164-1165, MT01167-1168), northern North Sea (60.3703°, 2.4784°), 100 m, 28.vii.2010, coll. H. Neumann. Two females (NHMUK unregistered), Shetland (North Sea), unknown water depth and collection date, coll. A.M. Norman. One adult female (NHMUK unregistered), Port Erin, Isle of Man (Irish Sea), unknown water depth, vii.1960, coll. I. Gordon. Pieces of three individuals (NHMUK 1952:5:7:152-155), Moray Firth (Scotland), unkown water depth and collection date, coll. Sp. Bate. Walker Collection (NHMUK 1925:9:8:877-887): One male, five females and four juveniles, Shiant and West Coast of Ireland, unkown water depth and collection date. Norman Collection (NHMUK 1911:11:8): One male and one female (16998–999), The Minch (Scotland), unknown water depth, 1866; diverse individuals (males, females, juveniles; 16934–943, 16914–933), Shetland (North Sea), unknown water depth, 1861; two females (16964–965), Clyde (Scotland), 1899; diverse individuals (males, females, juveniles; 16954–9563, 16975–976), unkown locality? (“Porcupine”), water depth and collection date. Approx. 100 specimens (males, females, juveniles; MCSN unregistered, “Rec Ledoyer sèr FVP 39”), south of Embiez (Toulon, France), 100–120 m, 26.vi.1975, coll. M. Ledoyer. Twenty individuals (males, females, juveniles; MCSN unregistered, “Rec Ledoyer Serie FVP, St. 33”), southeast of Canyon de Planir (south of Marseille, France), 90 m, 02.v.1975, coll. M. Ledoyer.

### Etymology

Named in honour of Prof. Heinz-Dieter Franke for his continuous support and inspiration of the first author (JB) for many years. The name *frankei* is the Latin genitive derived from his name.

### Diagnosis

Body dorsally smooth on pereonites 1–5; pereonite 6 roundly produced mid-dorsally; pereonite 7 with blunt mid-dorsal process; pleonites 1–3 strongly carinate, each with a blunt dorsolateral ridge; epimeral plates 1–3 biangular, with acute posterodistal teeth in epimera 2 and 3; urosomite 3 with distinctly upwards pointed projection; palp of maxilla 1 distally with 4 blunt spines and few subdistal setae; coxa 4 apical tip broadly rounded; coxa 5 anteroventral margin nearly straight or only slightly concave, apical tip of process broadly rounded; uropod 2 outer ramus distinctly shorter than inner ramus (about ¾).

### Description

Based on female holotype, 19 mm.

Body (Fig. 8A,B) dorsally smooth on pereonites 1–5; pereonite 6 roundly produced mid-dorsally; pereonite 7 with blunt mid-dorsal process and a blunt dorsolateral process. Pleonites 1–3 with blunt carinae and each with a blunt dorsolateral ridge; epimeral plates 1–3 biangular, produced to blunt tooth in epimeron 1 and acute posterodistal teeth in epimera 2 and 3, angles on posterior margins subacute. Urosomite 1 with blunt carina, notched mid-dorsally; urosomite 2 shortest; urosomite 3 with distinctly upwards pointed projection (Fig. 8D).

Head (Fig. 8C) about as high as long; ventral lobus slightly rounded, anterior corner rectangular. Rostrum weakly curved, ventral margin almost straight, about as long as 1^st^ peduncular article of antenna 1. Eyes large and rounded, bulging.

Antenna 1 (Fig. 9A) peduncular article 1 about 1.4 as long as articles 2–3 combined (length ratio 1: 0.5: 0.3); accessory flagellum consisting of a single tapering article, about 1/3 the length of the 1^st^ flagellar article; 1^st^ flagellar article about as long as peduncular article 2, primary flagellum with 30 articles.

Antenna 2 (Fig. 9B) slightly longer than antenna 1; peduncular article 1 shortest, article 2 partly overlapping articles 1 and 3; article 3 with distal cusp; article 4 slightly longer than article 5; flagellum with 38 articles.

Labrum (“upper lip”; Fig. 9D) ventrally rounded.

Hypopharynx (“lower lip”; Fig. 9C) with distally tapering lobes, densely coated with fields of setae on the medial margins.

Mandible (Fig. 9E) with 8-dentate incisor; lacinia mobilis triform (Fig. 9F); molar protruding, slender; mandibular palp (Fig. 9G) consisting of 3 articles, bearing long setae on the distal half of article 2 and the entire article 3 posteromarginally; setae length on palp article 3 increasing from proximal to distal.

Maxilla 1 (Fig. 10A) inner plate sub-triangular, with 11 stout plumose setae along the medial margin; outer plate truncate, with 11 mediodistally serrated spines (Fig. 10B); palp 2-articulate, article 2 about 5 times longer than the 1^st^ article, curved inwards, distally serrate with 4 blunt spines and few subdistal setae (Fig. 10B).

Maxilla 2 (Fig. 10C) inner lobe broader and shorter than outer lobe, with numerous setae along the medial margin; outer lobe with a row of slender spines which are mediodistally serrate along with few short slender setae.

Maxilliped (Fig. 10D,E) inner plate about 2/3 the length of 1^st^ palp article, apical ridge with blunt spines and stout setae; outer plate reaching about midpoint of palp article 2, ovoid, apical margin with progressively stouter, slender spines mediodistally, mediodistal region produced to a saw-like ridge; palp 4-articulate, medial margin of article 2 setose, article 3 with stout setae mediodistally, article 4 curved, with serrate medial margin.

Gnathopod 1 (Fig. 11A) coxa tapering distally and with broadly rounded apical tip, anterior margin nearly straight, posterior margin slightly concave and bearing a row of very fine setae; basis about as long as coxa, slightly curved anteriorly, posteromarginally with some long slender setae, setation of anterior margin sparse with few long slender setae proximally and a row of short slender setae mediodistally; ischium slightly wider than long; merus with longitudinal articulation of carpus, posterior margin oblique, with tuft of long setae posterodistally; carpus distally slightly expanded, with groups of long setae along the posterior margin and few short setae anterodistally; propodus about 0.8 of carpus length, posteriorly with tufts of setae; palm convex, with fine serration; dactylus curved, with serrate posterior margin.

Gnathopod 2 (Fig. 11B,C) coxa slightly longer than coxa 1, tapering distally with rounded apical tip, anterior margin sinuous, posterior margin distinctly convex medioapically with few very fine short setae mediomarginally; basis slightly shorter than coxa, nearly linear, distal half posteriomarginally with groups of long slender setae, sparse row of short setae along anterior margin with few long slender setae; ischium about as long as wide; merus and carpus of similar length and setation to gnathopod 1, but slightly narrower; propodus about as long as carpus, with tufts of setae along the posterior margin; palm obliquely convex, with fine serration and few setae on the lateral face (Fig. 11C); dactylus as for gnathopod 1.

Pereopod 3 (Fig. 12A) coxa subequal to coxa 2 but slightly longer and wider; basis about 0.7 as long as coxa, slightly sinuous but sublinear, posterior margin with groups of long slender setae on its distal half, anterior margin with sparse setation; ischium slightly longer than wide; merus length about 1.2 of carpus length, slightly curved posteriorly, with short setae along the posterior margin and tufts of setae antero- and posterodistally; carpus with tufts of stout setae posteromarginally; propodus about 1.2 the size of carpus, with 6 pairs of spine-like setae along the posterior margin; dactylus about half the size of propodus, curved.

Pereopod 4 (Fig. 12B) coxa strongly projecting ventrally and robust, anterior margin sinuous and strongly curved posteriorly, posterior margin excavate and with a centrally positioned rounded lobe, posteroapical margin strongly concave, apical tip broadly rounded; basis linear, posterior margin convex and with groups of long slender setae, anterior margin straight and with sparsely distributed slender setae; ischium about as long as wide, with a tuft of setae posterdistally; merus about 1.5 the size of carpus, sublinear but slightly curved posteriorly, with few short setae along the posterior margin and tufts of setae antero- and posterodistally; carpus with tufts of stout setae along the posterior margin; propodus about 1.2 the size of carpus, with 6 pairs of spine-like setae posteromarginally; dactylus about half the length of propodus, curved.

Pereopod 5 (Fig. 12C) coxa strongly extended posteroventrally, posterior margin evenly concave, anterior margin convex and describing a rounded right angle, anteroventral margin nearly straight, apical tip of process broadly rounded; basis rectangular, with posteriorly produced margin which expands to a bifid subrectangular lobe posterodistally, partly covering ischium when leg is retracted, anterior margin with long slender setae proximally and tufts of setae along the distal half; ischium longer than wide, lobate; merus about 1.2 the length of carpus, slightly curved anteriorly and slightly expanding distally, anterior margin with few very short setae, a single spine-like seta anterodistally and posterodistally; carpus slightly expanding distally, with 4 tufts of spine-like setae along anterior margin and posterodistally; propodus about 1.4 the size of carpus, with 5 pairs of spine-like setae anteromarginally; dactylus nearly half the size of propodus, curved.

Pereopod 6 (Fig. 13A) coxa subrectangular, longer than wide with an angular lateral lobe pointing posterodistally, anterior margin with fine setae; basis slightly curved posteriorly, with lobe roundly expanding proximally on the medial side of the posterior margin and posteriorly produced lateral margin which expands to a bifid subrectangular lobe distally, posteromedial lobe with row of short slender setae mediodistomarginally, anterior margin with long slender setae proximally and tufts of setae along the distal half; ischium longer than wide, lobate; merus about 1.2 the length of carpus, slightly curved anteriorly and slightly expanding distally, very short setae along the anterior margin and medioproximally on the posterior margin; carpus slightly expanding distally, with 4 tufts of stout setae anteromarginally; propodus about 1.3 the length of carpus, with 6 pairs of spine-like setae anteromarginally; dactylus large, more than half the length of propodus, curved.

Pereopod 7 (Fig. 13B,C) on right body side missing, except for the coxa; on left side all articles complete; coxa slightly longer than wide; basis much wider than pereopod 6, posteriorly lobate, posterodistal lobe subacute and partly covering ischium, with minute tooth anterodistally, anterior margin with few short setae; ischium about as long as wide, with minute tooth on the anterodistal margin; merus slightly longer than carpus, slightly curved anteriorly, anterior margin with pairs of short stout setae, posterodistal corner bearing a blunt spine; carpus with 4 tufts of stout setae anteromarginally; propodus 1.3 the length of carpus, with 6 groups of spine-like setae anteromarginally; dactylus about half the length of propodus, curved.

Uropod 1 (Fig. 13D) peduncle subrectangular, with small spines along medial and lateral margins, spines increasing in size from proximal to distal; rami subequal, about 1.2 the length of peduncle, margins bordered with small spines.

Uropod 2 (Fig. 13E) peduncle weakly expanding distally, lateral margin with row of small spines; outer ramus distinctly shorter than inner ramus (about ¾), inner ramus about double as long as peduncle, spination similar to rami of uropod 1.

Uropod 3 (Fig. 13F) peduncle shortest, with small spines along the sublateral margin, and short setae on the medial margin; rami subequal in length, spination similar to rami of uropod 1 and 2.

Telson (Fig. 13G) about 1.5 as long as wide, slightly notched to only 10% of its length, apices broadly rounded.

### Variation

In general, males of *E*. *frankei* resemble their females except for smaller body sizes. They can, however, be distinguished from the females by some distinct morphological features of the trunk (Fig. 13H). In direct comparison, the female pereon segments are clearly broader than in males, which is particularly visible in dorsal view. Males, in return, tend to feature a proportionally more pronounced pleon. Furthermore, male coxal plates tend to be narrower and less tubby/rounded, resulting in a smaller coxal surface area. There is little intraspecific variation except for the sexual dimorphism described above. In the examined material, pereon segments 6–7 and pleon segments of specimens from the Mediterranean, however, exhibited higher mid-dorsal processes (Fig. 13I).

**Figure 14:**
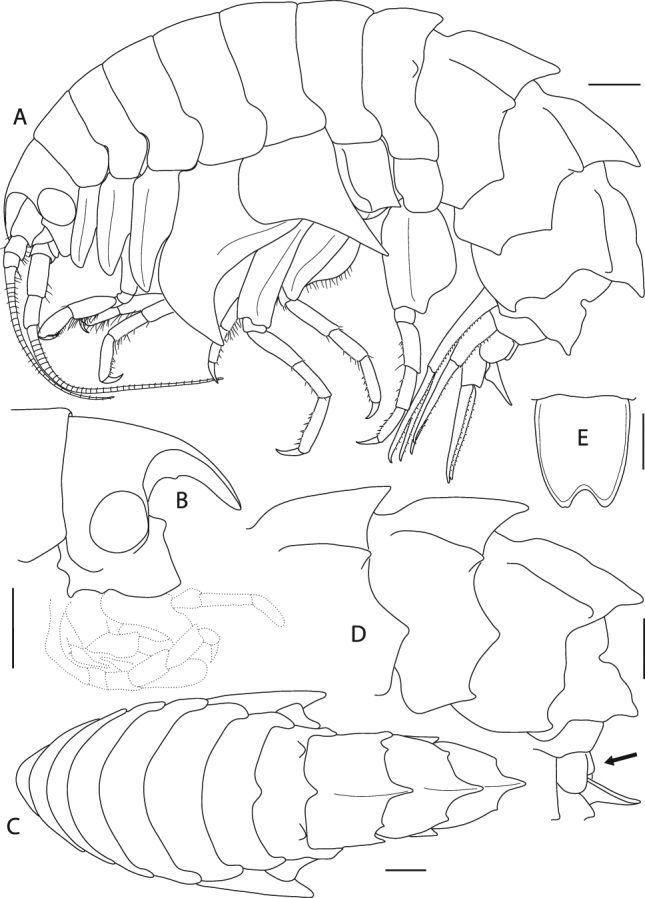
*Epimeria cornigera*, neotype, adult female, northern North Sea, NHMUK 2015.3268: (**A**) lateral habitus, (**B**) head in lateral view, (**C**) dorsal habitus, (**D**) pleon and urosome, (**E**) telson; scales: (A,B,C,D) 1 mm; (E) 0.5 mm.

**Figure 15:**
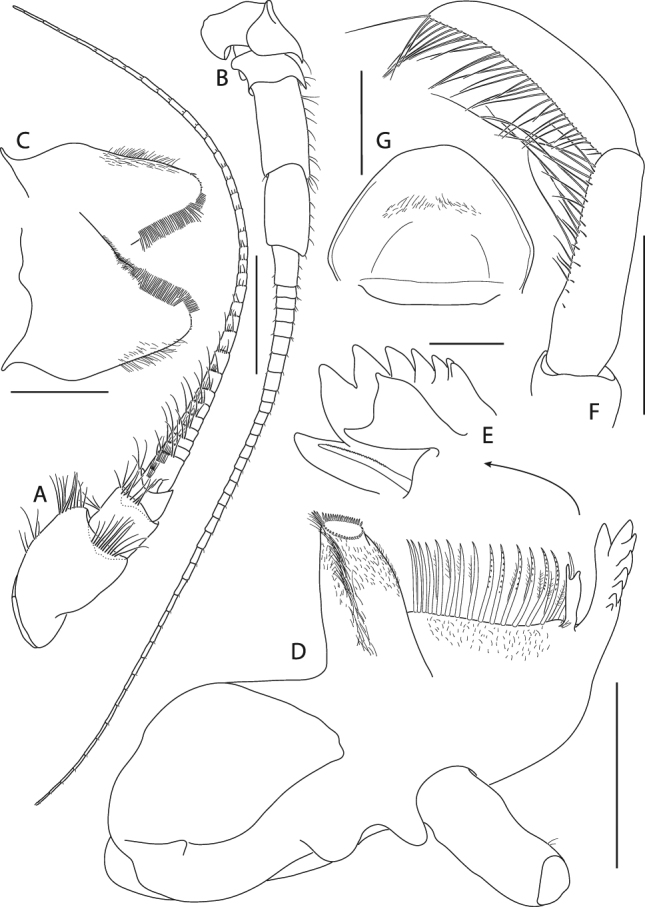
*Epimeria cornigera*, neotype, adult female, northern North Sea, NHMUK 2015.3268: (**A**) antenna 1, (**B**) antenna 2, (**C**) hypopharynx, (**D**) mandible, (**E**) mandible incisor and lacina mobilis, (**F**) mandibular palpus, (**G**) labrum; scales: (A,B) 1 mm; (C,D,F,G) 0.5 mm; (E) 0.1 mm.

**Figure 16:**
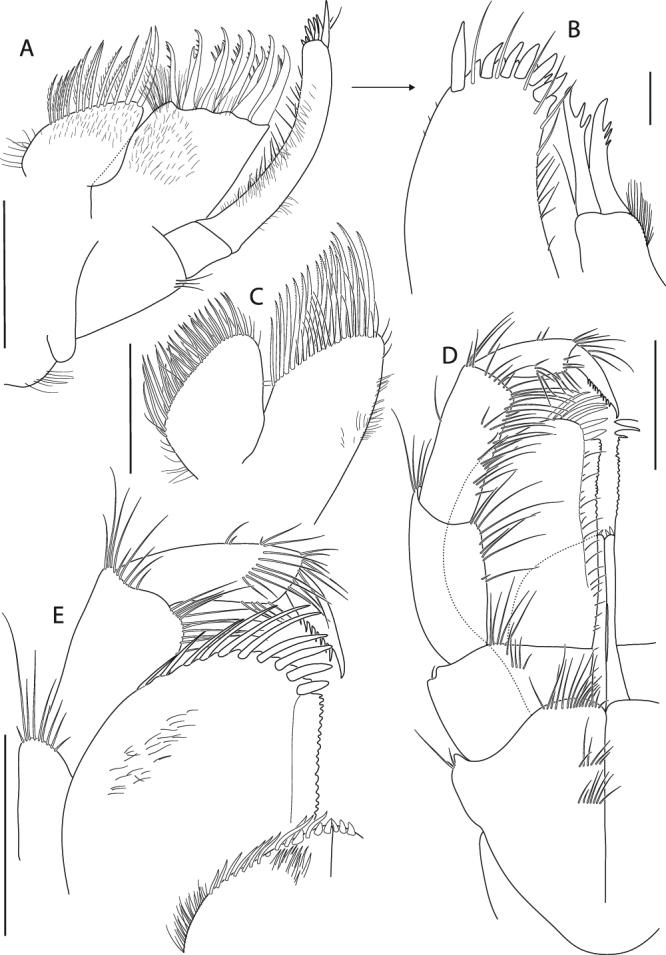
*Epimeria cornigera*, neotype, adult female, northern North Sea, NHMUK 2015.3268: (**A**) maxilla 1, (**B**) maxilla 1 palpus and outer plate distal ends lateral view, (**C**) maxilla 2, (**D**) maxilliped, (**E**) maxilliped palpus and outer plate; scales: (A,C,D,E) 0.5 mm; (B) 0.1 mm.

**Figure 17:**
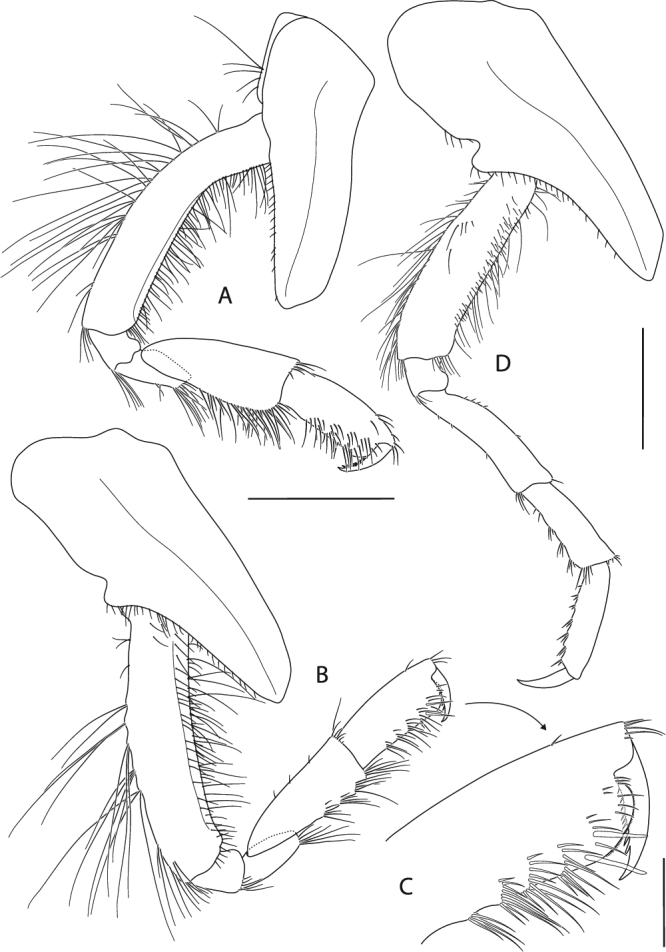
*Epimeria cornigera*, neotype, adult female, northern North Sea, NHMUK 2015.3268: (**A**) gnathopod 1, (**B**) gnathopod 2, (**C**) distal articles of gnathopod 2, (**D**) pereopod 3; scales: (A,B,D) 1 mm; (C) 0.25 mm.

**Figure 18:**
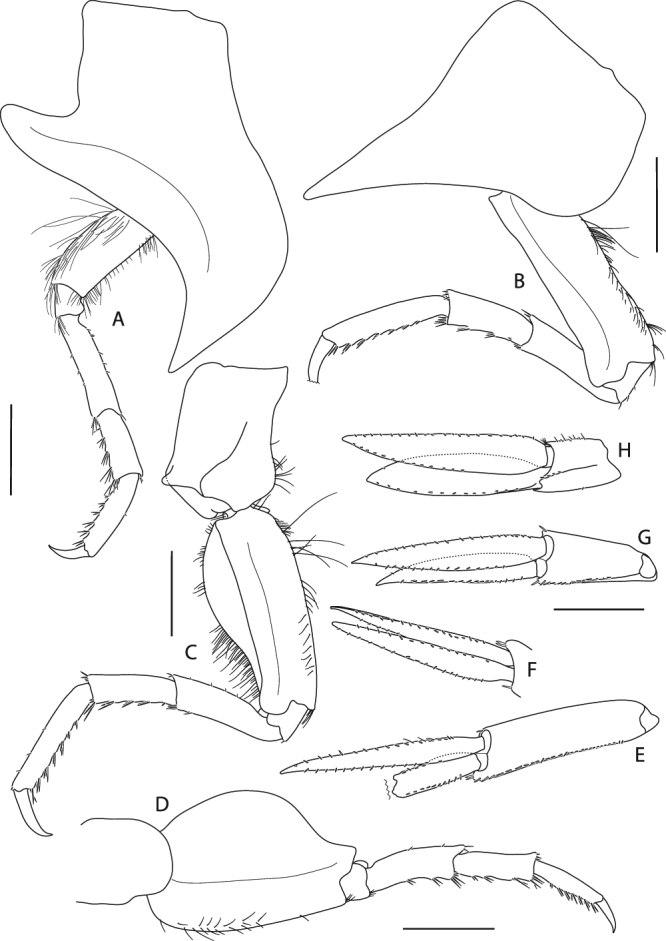
*Epimeria cornigera*, neotype, adult female, northern North Sea, NHMUK 2015.3268: (**A**) pereopod 4, (**B**) pereopod 5, (**C**) pereopod 6, (**D**) left pereopod 7 (drawn on habitus), (**E**) uropod 1 (outer ramus damaged), (**F**) rami of left uropod 1 (drawn on habitus), (**G**) uropod 2, (**H**) uropod 3; all scales 1 mm.

**Figure 19:**
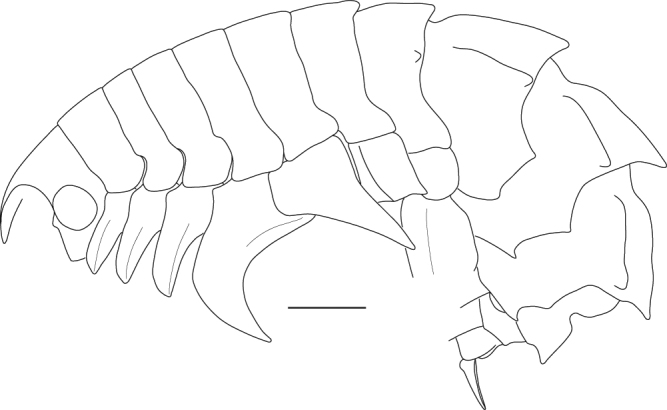
*Epimeria cornigera*, paratype, adult male, northern North Sea, NHMUK 2015.3269, lateral habitus; scale 1 mm.

***Epimeria cornigera*** **(Fabricius**, **1779)** (Figs 14, 15, 16, 17, 18 and 19)

*Gammarus corniger* Fabricius^113^, 1779: 383.

*Epimeria tricristata* Costa^114^, 1851: 46.

*Acanthonotus owenii* Bate^115^, 1857: 141. – Bate & Westwood^116^, 1863.

*Acanthonotus testudo* Bate^117^, 1862: 127.

*Epimeria cornigera* Boeck^118^, 1871: 185. – Sars^55^, 1893 364, pl 128. – Stebbing^119^, 1906: 323. – Stephensen^56^, 1938: 266. – Lincoln^120^, 1979: 436, fig. 207, 208a.

### Type material examined

Holotype lost, type locality presumed as “coast of Norway”. Neotype (NHMUK 2015.3268), ovigerous female, 18 mm, northern North Sea – approx. 150 km west of Bergen (60.3104°, 2.4967°) in 97 m water depth, 05.viii.2013, coll. H. Neumann. Paratypes: two adult males, 14 mm (Fig. 19; NHMUK 2015.3269) and 14.5 mm (NHMUK 2015.3270) and seven adult females (6 ovigerous; NHMUK 2015.3271 − 3277), locality data identical to holotype.

### Other material

Five males, three adult females (1 ovigerous) and one juvenile female (MT06700, MT06716-06717, MT06765-06769, MT06786), western central North Sea (57.394°, 0.2925°), 78 m, 10.iii.2012, coll. H. Neumann. One adult female (MT06718), western North Sea (54.8732°, −0.9581°), 68 m, 07.iii.2012, coll. H. Neumann. One male (MT06709), northern North Sea (60.3659°, 2.4386°), 96 m, 31.vii.2012, coll. I. Rottgardt. One adult female (MT06705), northern North Sea (60.3583°, 2.5371°), 106 m, 01.viii.2012, coll. I. Rottgardt. One ovigerous female (MT06702) western North Sea (55.1097°, −0.5023°), 82 m, 07.ii.2012, coll. H. Neumann. Two adult females (1 ovigerous; MT01169-01170), northern North Sea (60.3703°, 2.4784°), 100 m, 28.vii.2010, coll. H. Neumann. Two adult females (NHMUK 1985:233:2), North Sea (57.6000°, 0.5733°), 88–94 m, 1985, unknown collector. Five males, ten females (5 ovigerous) and 28 juveniles (NHMUK 1984:566:30), Irish Sea (Cumbrae Elbow.), 18 m (10 fathoms), 08.viii.1984, coll. R.J. Lincoln and G.A. Boxshall. One ovigerous female (NHMUK 1907:12:2:122), Irish Sea (Mull of Cantyre), unknown water depth and collection date, coll. University College of Dundee. One male and three ovigerous females (NHMUK 1887.21), Scottish coast? (“Loche Lyne”), unkown water depth and collection date, coll. J. Murray. Two ovigerous females (NHMUK 1986:606:2), North Sea (57.6050°, 0.5716°), 89 m, 24.viii.1985, coll. G. Cranmer. Norman Collection (NHMUK 1911:11:8): One male and four ovigerous females (16981-995), Trondheimsfjord (Norway), 365 m (200 fathoms), 1869; two adult females (16977-980), Hardangerfjord (Norway), 274–329 m, unknown collection date; ten adult females (16944-953), Durham Coast (western North Sea), unkown water depth and collection date. One adult female (MCSN 262), Irish Sea (53.7833°, −4.9666°), 80 m, 17.viii.1992, unknown collector. One ovigerous female (MCSN Z8-67), Bømlafjorden (Norway), water depth, sampling date and collector unknown. One male and one ovigerous female (MCSN 11177), Hjeltefjorden (60.4050°, 5.1169°), water depth and collection date unknown, coll. W. Vader.

### Diagnosis

Body dorsally smooth on pereonites 1–5; pereonite 6 not produced or only slightly produced mid-dorsally; pereonite 7 with mid-dorsal process; pleonites 1–3 strongly carinate, each with a blunt dorsolateral ridge; epimeral plates 1–3 biangular, produced to subacute posterodistal teeth in epimera 2 and 3; urosomite 3 with only slightly produced rounded hump; palp of maxilla 1 distally with 6 blunt spines and few subdistal setae; coxa 4 apical tip subacute; coxa 5 anteroventral margin distinctly or strongly concave, apical tip of process subacute; uropod 2 outer ramus only slightly shorter than inner ramus (about 4/5).

### Description

Based on female neotype, 18 mm.

Body (Fig. 14A,C) dorsally smooth on pereonites 1–5; pereonite 6 slightly produced mid-dorsally; pereonite 7 with mid-dorsal process and a rounded dorsolateral process posteriorly. Pleonites 1–3 strongly carinate, each with a blunt dorsolateral ridge; epimeral plates 1–3 biangular, produced to subacute posterodistal teeth on epimera 2–3 and broadly rounded on epimeron 1, angles on posterior margins blunt to broadly rounded. Urosomite 1 with apically rounded carina, notched mid-dorsally; urosomite 2 shortest; urosomite 3 with slightly produced rounded hump (Fig. 14D).

Head (Fig. 14B) about as high as long; ventral lobus slightly rounded, anterior corner rectangular. Rostrum curved, ventral margin rounded, about as long as 1^st^ peduncular article of antenna 1. Eyes large and rounded, bulging.

Antenna 1 (Fig. 15A) peduncular article 1 about 1.5 the length of articles 2–3 combined (length ratio 1: 0.6: 0.3); accessory flagellum consisting of a single tapering article, about 1/3 the length of the 1^st^ flagellar article; 1^st^ flagellar article about as long as peduncular article 2, primary flagellum with 36 articles.

Antenna 2 (Fig. 15B) slightly longer than antenna 1; peduncular article 1 about as long as article 3, article 2 partly overlapping articles 1 and 3, with distal cusp; article 3 with distal cusp; article 4 slightly longer than article 5; flagellum with 39 articles.

Labrum (“upper lip”; Fig. 15G) ventrally rounded.

Hypopharynx (“lower lip”; Fig. 15C) with distally tapering lobes, densely coated with fields of setae on the medial margins.

Mandible (Fig. 15D) with 8-dentate incisor; lacinia mobilis bifid (Fig. 15E); molar protruding and slender; mandibular palp (Fig. 15F) consisting of 3 articles, bearing long setae on the distal half of article 2 and the entire article 3 posteromarginally, setae length on palp article 2 and 3 increasing from proximal to distal.

Maxilla 1 (Fig. 16A) inner plate sub-triangular, with 13 stout plumose setae along the medial margin; outer plate truncate, with 11 mediodistally serrated spines (Fig. 16B); palp 2-articulate, article 2 about 6 times longer than the 1^st^ article, curved inwards, distally serrate with 6 blunt spines and few subdistal setae (Fig. 16B).

Maxilla 2 (Fig. 16C) inner lobe slightly broader and shorter than outer lobe, with numerous setae along the medial margin; outer lobe with a row of slender spines which are mediodistally serrate along with few short slender setae.

Maxilliped (Fig. 16D,E) inner plate about 2/3 the length of 1^st^ palp article, apical ridge with blunt spines and stout setae; outer plate reaching about midpoint of palp article 2, ovoid, apical margin with slender spines which get stouter mediodistally, mediodistal region produced to a saw-like ridge; palp 4-articulate, mediodistal margin of article 1 with long setae, medial margin of article 2 strongly setose, article 3 with setae medio- and laterodistally, article 4 curved, with serrate medial margin.

Gnathopod 1 (Fig. 17A) coxa tapering distally and having a rounded subangular apical tip, anterior margin sinuous, posterior margin straight to slightly convex and bearing a row of short very fine setae along medioapically as well as longer setae subproximally; basis about as long as coxa, distinctly curved anteriorly, posteromarginally with tufts of long slender setae, anterior margin strongly setose with fine setae along its entire length; ischium slightly longer than wide, with tuft of setae posterodistally; merus with longitudinal articulation of carpus, posterior margin oblique, with group of setae posterodistally; carpus distally slightly expanded, with dense setation along the posterior margin and a tuft of shorter setae anterodistally; propodus about 0.8 of carpus length, posteriorly with setae along the margin and on the lateral face; palm convex, with fine serration; dactylus curved, with serrate posterior margin.

Gnathopod 2 (Fig. 17B,C) coxa slightly longer than coxa 1, tapering distally with a rounded subangular tip, anterior margin slightly sinuous, posterior margin distinctly convex medioapically with a row very fine short setae along the margin; basis slightly shorter than coxa, slightly curved anteriorly, posteromarginally with tufts of long slender setae, long slender setae along the entire anterior margin accompanied with few shorter slender setae; ischium slightly longer than wide, with group of setae posterodistally; merus similar in length and setation to gnathopod 1; carpus shape similar to gnathopod 1, with tufts of long setae on the posterior margin; propodus about 0.8 the length of carpus, with transverse rows of setae along the posterior margin; palm obliquely convex, with fine serration and 2 long blunt spines subdistally on the lateral face (Fig. 17C); dactylus as for gnathopod 1.

Pereopod 3 (Fig. 17D) coxa subequal to coxa 2 but slightly longer and wider; basis about 0.8 as long as coxa, sublinear but slightly curved anteriorly, posterior margin with tufts of long slender setae, anterior margin with long slender setae and a field of very short fine setae which extends to the lateral face; ischium about as long as wide; merus length about 1.3 of carpus length, slightly curved posteriorly and distally expanding, with few short setae at the posterior and anterior margins as well as groups of setae antero- and posterodistally; carpus slightly expanding distally, with tufts of stout setae posteromarginally; propodus about 1.1 the size of carpus, with 7 pairs of spine-like setae along the posterior margin; dactylus about half the size of propodus, curved.

Pereopod 4 (Fig. 18A) coxa strongly projecting ventrally and robust, anterior margin sinuous and strongly curved posteriorly, posterior margin excavate and with a centrally positioned rounded lobe, posteroapical margin strongly concave, apical tip subacute; basis linear, posterior margin convex and with long slender setae extending to the lateral face, anterior margin straight and with shorter slender setae; ischium slightly longer than wide, with a group of setae posterdistally; merus about 1.5 the size of carpus and sublinear, with few short setae along the margins and groups of setae antero- and posterodistally; carpus with tufts of stout setae along the posterior margin; propodus about 1.3 the size of carpus, with 6 pairs of spine-like setae posteromarginally; dactylus less than half the length of propodus, curved.

Pereopod 5 (Fig. 18B) coxa strongly extended posteroventrally, posterior margin slightly concave, anterior margin convex and strongly curved posteriorly, anteroventral margin distinctly concave, apical tip of process subacute; basis rectangular, with posteriorly produced margin which expands to a bifid subrectangular lobe posterodistally, partly covering ischium when leg is retracted, anterior margin with numerous setae along its entire length; ischium longer than wide, lobate; merus about 1.4 the length of carpus, slightly curved anteriorly, with short stout setae postero- and anterodistally; carpus slightly expanding distally, with tufts of spine-like setae along the anterior margin and posterodistally; propodus about 1.5 the size of carpus, with 7 tufts of spine-like setae anteromarginally; dactylus on right pereopod damaged, but clearly less than half the size of propodus, curved.

Pereopod 6 (Fig. 18C) coxa subrectangular, longer than wide with a laterally protruding ridge anteriorly and an angular lateral lobe pointing posterodistally, anterior margin with fine setae; basis slightly curved posteriorly, with lobe roundly expanding proximally on the medial side of the posterior margin and posteriorly produced lateral margin which expands to a bifid subrectangular lobe distally, posteromedial lobe with many slender setae mediodistomarginally, anterior margin with long slender setae proximally and few setae along the distal half; ischium longer than wide, lobate; merus about 1.3 the length of carpus, slightly curved anteriorly and slightly expanding distally, with very short setae along the posterior margin and tufts of setae postero- and anterodistally; carpus slightly expanding distally, with 5 groups of stout setae anteromarginally; propodus about 1.5 the length of carpus, with 7 groups of spine-like setae anteromarginally; dactylus about half the length of propodus, curved.

Pereopod 7 (Fig. 18D) on right body side missing; on left side all articles complete; coxa longer than wide; basis much wider than pereopod 6, posteriorly lobate, posterodistal lobe subacute and partly covering ischium, anterior margin with few short setae; ischium about as long as wide; merus slightly longer than carpus, slightly curved anteriorly, anterior margin with groups of short stout setae, posterodistal corner bearing two setae; carpus with 4 groups of stout setae anteromarginally; propodus about as long as carpus, with 7 groups of spine-like setae anteromarginally; dactylus about half the length of propodus, curved.

Uropod 1 (Fig. 18E,F) on right body side with damaged outer ramus, but undamaged on left body side; peduncle subrectangular, with small spines along medial margin, spine size slowly increasing from proximal to distal; rami subequal, about 1.2 the length of peduncle, margins bordered with small spines.

Uropod 2 (Fig. 18G) peduncle expanding distally, lateral margin with row of small spines; outer ramus slightly shorter than inner ramus (about 4/5), inner ramus about 1.7 the length of peduncle, spination similar to rami of uropod 1.

Uropod 3 (Fig. 18H) peduncle shortest, with small spines along the sublateral margin, and short setae on the medial margin; outer ramus slightly shorter than inner ramus, spination similar to rami of uropod 1 and 2.

Telson (Fig. 14E) about 1.5 as long as wide, slightly notched to about 15% of its length, apices pointedly rounded.

### Variation

Similar to *E*. *frankei*, males of *E*. *cornigera* resemble their females, except for smaller body sizes. The described morphological differences between the sexes in *E*. *frankei* (see above) also apply to *E*. *cornigera* (Fig. 19).

## Electronic supplementary material

Supplementary Information
