# Supplementary material for: Cryptic species in a well-known habitat: applying taxonomics to the amphipod genus Epimeria (Crustacea, Peracarida)
Source: Sci Rep. 2018 May 2;8:6893. doi: 10.1038/s41598-018-25225-x (PMC5931980; doi:10.1038/s41598-018-25225-x)
Supplement: Supplementary file 1 — Supplementary Information [file 41598_2018_25225_MOESM1_ESM.docx]

**Cryptic species in a well-known habitat: applying taxonomics to the amphipod genus *Epimeria* (Crustacea, Peracarida)**

Jan Beermann, Michael V. Westbury, Michael Hofreiter, Leon Hilgers, Fabian Deister, Hermann Neumann, Michael J. Raupach

Table S1: Position and location of substitutions between the 18S rRNA genes of *Epimeria cornigera* and *Epimeria frankei* sp. nov..

| **No.** | **Alignmentposition** | ***Epimeria cornigera*** | ***Epimeria frankei*** | **18S rRNA Region** |
| --- | --- | --- | --- | --- |
| 1 | 80 | A | G | V1 |
| 2 | 141 | A | C |  |
| 3 | 210 | A | G | V2 |
| 4 | 269 | G | C | V2 |
| 5 | 274 | T | C | V2 |
| 6 | 279 | A | T | V2 |
| 7 | 280 | G | T | V2 |
| 8 | 419 | C | T |  |
| 9 | 426 | T | C |  |
| 10 | 465 | C | T |  |
| 11 | 824 | T | C | V4 |
| 12 | 825 | C | T | V4 |
| 13 | 940 | C | A | V4 |
| 14 | 942 | T | C | V4 |
| 15 | 944 | T | G | V4 |
| 16 | 946 | C | T | V4 |
| 17 | 953 | C | T | V4 |
| 18 | 954 | T | G | V4 |
| 19 | 1009 | G | T | V4 |
| 20 | 1010 | G | T | V4 |
| 21 | 1097 | T | C | V4 |
| 22 | 1229 | C | G | V4 |
| 23 | 1230 | G | T | V4 |
| 24 | 1231 | C | T | V4 |
| 25 | 1846 | T | C | V7 |
| 26 | 1904 | A | G | V7 |
| 27 | 1936 | A | G | V7 |
| 28 | 1941 | T | C | V7 |
| 29 | 1943 | T | C | V7 |
| 30 | 1946 | C | T | V7 |
| 31 | 1949 | G | A | V7 |
| 32 | 2021 | A | G | V7 |
| 33 | 2234 | T | C |  |
| 34 | 2239 | C | T |  |

Table S2: Summary of all used *Epimeria* species downloaded from GenBank for the Maximum Likelihood analysis, including the number of sequences, corresponding accession numbers, and references.

| **Taxon** | ***n*** | **Accession numbers** | **Reference** |
| --- | --- | --- | --- |
| *Epimeria (Urepimeria) annabellae* Coleman, 1994 | 1 | ^$^FM955293 | ^$^Lörz et al. 2009 |
| *Epimeria (Hoplepimeria) angelikae* (Lörz & Linse, 2011) | 2 | ^$^FM955305, ^$^FM955299 | ^$^Lörz et al. 2009 |
| *Epimeria bruuni* J.L. Barnard, 1961 | 1 | ^$^FM955298 | ^$^Lörz et al. 2009 |
| *Epimeria (Hoplepimeria) cyphorachis* d'Udekem d'Acoz & Verheye, 2017 | 7 | AY061802, ^§^JF271110, ^§^JF271111, ^§^JF271112, ^§^JF271113, ^§^JF271114, ^§^JF271115 | Lörz & Held 2004, ^§^Lörz et al. 2012 |
| *Epimeria (Pseudepimeria) cf. grandirostris* (Chevreux, 1912) | 1 | ^$^FM955307 | ^$^Lörz et al. 2009 |
| *Epimeria horsti* Lörz, 2008 | 2 | ^$^FM955296, ^$^FM955297 | ^$^Lörz et al. 2009 |
| *Epimeria (Hoplepimeria)* *inermis* Walker, 1903 | 6 | ^$^FM955280, ^$^FM955281, ^$^FM955282, ^$^FM955285, ^$^FM955292, ^§^JF271116 | ^$^Lörz et al. 2009, ^§^Lörz et al. 2012 |
| *Epimeria (Hoplepimeria) linseae* d'Udekem d'Acoz & Verheye, 2017 | 4 | ^§^JF271105, ^§^JF271106, ^§^JF271107, ^§^JF271109 | ^§^Lörz et al. 2012 |
| *Epimeria (Drakepimeria) cf. macrodonta* Walker, 1906 | 1 | AF451343 | Lörz & Held 2004 |
| *Epimeria (Epimeriella) macronyx* (Walker, 1906) | 1 | ^$^FM955309 | ^$^Lörz et al. 2009 |
| *Epimeria (Hoplepimeria) quasimodo* d'Udekem d'Acoz & Verheye, 2017 | 4 | AF451341, , ^§^JF271102, ^§^JF271103, ^§^JF271104, | Lörz & Held 2004, ^§^Lörz et al. 2012 |
| *Epimeria (Subepimeria) cf. puncticulata* K.H. Barnard, 1930 | 1 | ^$^FM955301 | ^$^Lörz et al. 2009 |
| *Epimeria (Drakepimeria) reoproi* (Lörz & Coleman, 2001) | 1 | AF451342 | Lörz & Held 2004 |
| *Epimeria (Hoplepimeria) rimicarinata* (Watling & Holman, 1980) | 7 | ^$^FM955300, ^$^FM955302, ^$^FM955303, ^$^FM955304, ^#^GU804288, ^§^JF271117, ^§^JF271118 | ^$^Lörz et al. 2009, ^§^Lörz et al. 2012, ^#^iBOL data release 2016 |
| *Epimeria (Hoplepimeria) robustoides* (Lörz & Coleman, 2009) | 9 | AF451344, ^$^FM955279, ^$^FM955283,  ^$^FM955286, ^$^FM955287, ^$^FM955288,  ^$^FM955289, ^$^FM955290, ^$^FM955291, | Lörz & Held 2004, ^$^Lörz et al. 2009 |
| *Epimeria (Hoplepimeria) rubrieques* (De Broyer & Klages, 1991) | 1 | AF451345 | Lörz & Held 2004 |
| *Epimeria (Drakepimeria) schiaparelli* (Lörz, Maas, Linse & Fenwick, 2007) | 12 | ^¥^AM176763, ^¥^AM176764, ^¥^AM176765, ^¥^AM176767, ^¥^AM176768, ^¥^AM176769, ^¥^AM176770, ^¥^AM176771, ^¥^AM176772, ^¥^AM176773, ^¥^AM398942, ^$^FM955284 | ^¥^Lörz et al. 2007, ^$^Lörz et al. 2009 |
| *Epimeria (Drakepimeria) cf. similis* (Chevreux, 1912) | 1 | AF451346 | Lörz & Held 2004 |
| *Epimeria* sp. | 1 | ^$^FM955295 | ^$^Lörz et al. 2009 |
| *Epimeria (Laevepimeria) cf. walkeri* (K.H. Barnard, 1930) | 2 | ^$^FM955306, ^$^FM955308 | ^$^Lörz et al. 2009 |

**References:**

Lörz AN, Held C (2004) A preliminary molecular and morphological phylogeny of the Antarctic Epimeriidae and Iphimediidae (Crustacea, Amphipoda). Molecular Phylogenetics and Evolution 31: 4-15.

Lörz AN, Maas EW, Linse K, Fenwick GD (2007) *Epimeria schiaparelli* sp. nov., an amphipod crustacean (family Epimeriidae) from the Ross Sea, Antarctica, with molecular characterisation of the species complex. Zootaxa 1402: 23-37.

Lörz AN, Maas EW, Linse K, Coleman CO (2009) Do circum-Antarctic species exist in peracarid Amphipoda: a case study in the genus *Epimeria* Costa, 1851 (Epimeriidae). Zookeys 18: 91-128.

Lörz AN, Smith P, Linse K, Steinke D (2012) High genetic diversity within *Epimeria georgiana* (Amphipoda) from the southern Scotia Arc. Marine Biodiversity 42: 137-159.
